# Plasma proenkephalin‐a facilitates measurement of human kidney functional reserve: From healthy kidney donors to polymorbid patients

**DOI:** 10.14814/phy2.71083

**Published:** 2026-08-26

**Authors:** Camila Eleuterio Rodrigues, Amy Y. M. Au, Daniel Rebello, Maria Maawad, Florian Uhle, Jonathan Erlich, Zoltan H. Endre

**Affiliations:** ^1^ School of Clinical Medicine, Faculty of Medicine and Health University of New South Wales Sydney New South Wales Australia; ^2^ Department of Nephrology Prince of Wales Hospital Sydney New South Wales Australia; ^3^ Hospital das Clínicas da Faculdade de Medicina da Universidade de São Paulo São Paulo SP Brazil; ^4^ SphingoTec GmbH Hennigsdorf Germany

**Keywords:** creatinine secretion, cystatin C, kidney functional reserve, proenkephalin‐A, subclinical chronic kidney disease

## Abstract

Reduced kidney functional reserve, KFR, predicts poor outcomes in kidney diseases. We compared plasma proenkephalin‐A, plasma cystatin C, and creatinine clearance for kidney functional reserve measurement. KFR was measured preoperatively in 40 patients undergoing cardiac surgery and 22 healthy participants, including 14 kidney donors. Plasma proenkephalin‐A, cystatin C, and creatinine levels were compared before and after a protein meal for KFR calculation. Baseline creatinine clearance correlated poorly with baseline estimated glomerular filtration rate measured by any method. Cystatin C and proenkephalin‐A nadir after protein occurred earlier. The proenkephalin‐A‐based KFR had a greater dynamic range than cystatin C‐ and creatinine clearance‐based KFR (median [IQR] fractional KFR: proenkephalin‐A 44.2% [35.5; 58.0]; cystatin C 10.5% [4.4; 16.2]; creatinine clearance 30.0% [8.4; 68.9], *p* < 0.0001). Measurements were more homogeneous with cystatin C‐ and proenkephalin‐A‐estimated glomerular filtration rate than creatinine clearance (higher intraclass correlation: 0.98 for cystatin C; 0.86 for proenkephalin‐A, and 0.63 for creatinine clearance). The median [IQR] absolute proenkephalin‐A‐ and cystatin C‐based KFR were higher in healthy participants (proenkephalin‐A‐KFR: 33.8 [26.6; 59.0], cystatin C‐KFR: 11.6 [6.6; 15.1] mL/min/1.73m^2^) than cardiac surgery patients (proenkephalin‐A‐reserve: 28.5 [19.9; 40.6], cystatin C‐reserve: 5.0 [0.3; 8.7] mL/min/1.73m^2^, *p* < 0.05). Creatinine clearance‐KFR did not differ between groups and mostly reflected tubular secretion of creatinine than change in glomerular filtration rate. In conclusion, proenkephalin‐A provided a brief, homogeneous measure of KFR with wide dynamic range, encouraging wider use of this kidney stress test.

## Graphical abstract

Enhanced measurement of Kidney Functional Reserve (KFR) by biomarkers: Protein ingestion stimulates intrarenal blood flow and increases glomerular filtration rate (GFR). KFR is [stimulated – baseline] GFR and can be assessed by biomarkers of filtration, including plasma proenkephalin‐A, plasma cystatin C, and creatinine clearance. Hyperfiltering nephrons lose capacity to further increase single nephron GFR, so that reduced KFR identifies subclinical chronic kidney disease. Proximal tubular creatinine secretion contributes significantly to creatinine clearance. KFR measured by plasma proenkephalin‐A or cystatin C identifies GFR reserve more rapidly and is not affected by changes in tubular creatinine secretion.

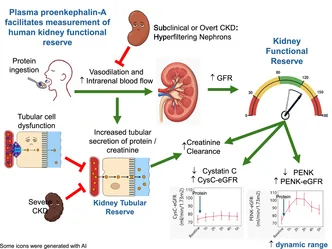

## 1 INTRODUCTION

Stress tests of kidney function are infrequent in clinical practice but can detect underlying subclinical kidney disease despite a normal plasma creatinine (pCr). Kidney functional reserve (KFR) is defined as the increase in glomerular filtration rate (GFR) after a physiological stimulus, such as pregnancy, and is most commonly assessed after an oral protein load (Cassidy & Beck, 1988; Chan, 1986; Herrera & Rodríguez‐Iturbe, 1998; Husain‐Syed et al., 2018; Rodríguez‐Iturbe et al., 1985; van Londen et al., 2018).

Reduced KFR may parallel hyperfiltration in the remaining nephrons after incomplete recovery from injury in kidney disease (Hostetter et al., 1981). When measured directly by fluorescein isothiocyanate (FITC)‐labeled sinistrin in rodents (Taylor et al., 2023) or clearance of Tc99m‐DTPA in humans (Barai et al., 2010), KFR detects subclinical chronic kidney disease (sCKD) and predicts chronic kidney disease (CKD) progression and mortality. We previously showed KFR assessed by plasma cystatin C (CysC) is equivalent to KFR measured by technetium‐99 m‐diethlenetriamine pentaacetate (Tc99m‐DTPA) and facilitates KFR determination, predicting death, dialysis, and major adverse kidney events in CKD (Christiadi et al., 2022). CysC‐KFR also predicts postoperative Acute Kidney Injury (AKI) following cardiopulmonary bypass (Fuhrman et al., 2025). Proenkephalin‐A (PENK) is more accurate than other markers in non‐steady‐state conditions for estimating GFR (Khorashadi et al., 2020) and detects AKI earlier than other plasma kidney functional biomarkers (Beunders et al., 2020, 2023; Smeets et al., 2023), but has never been used to assess KFR.

Decreased preoperative creatinine clearance (CrCl)‐KFR predicts postoperative AKI after cardiac surgery (Husain‐Syed et al., 2018) and incomplete kidney recovery after hematopoietic stem cell transplantation‐associated AKI (Mancianti et al., 2023), suggesting prior sCKD in those who developed AKI. However, CrCl measurement is difficult, which makes large‐scale application challenging, is potentially inaccurate in non‐catheterised subjects, and includes tubular secretion as well as glomerular clearance of creatinine (Herrera & Rodríguez‐Iturbe, 1998).

KFR loss occurs in kidney donors with hypertension, older age, or obesity (Figurek et al., 2020). Nevertheless, clinical implications remain uncertain, including whether recruitment of KFR allows recovery of function after kidney donation.

This study compared KFR measurement by plasma PENK with CysC and CrCl in healthy subjects and prior to cardiac surgery. We assessed time to plasma nadir and dynamic range and hypothesized that PENK‐based KFR would reduce the duration of KFR assessment. As a secondary aim, we compared pre‐ and postoperative KFR in healthy kidney donors and hypothesized that postoperative GFR in donor kidneys improved by recruitment of KFR, placing donors at possible risk of future reductions in function.

## 2 MATERIALS AND METHODS

### 2.1 Study recruitment

In this prospective observational study, KFR was measured pre‐ and postoperatively in adult patients undergoing unilateral nephrectomy for kidney donation or auto‐transplantation in the “Preoperative Kidney Functional Reserve Assessment in Patients Undergoing Nephrectomy as a PREDICTor of Long‐Term Renal Outcomes” (PREDICT) Study and preoperatively in adults undergoing elective cardiac surgery in the “Pre‐operative kidney functional REServe and damage biomarkers in PrEdicTion of perioperative acute kidney injury (AKI) after cardiac surgery” (RESPECTAKI) Study. KFR was also measured in healthy volunteers. Individual studies were approved by the South Eastern Sydney Local Health District Clinical Ethics Committee (HREC/2021/ETH11453 and 2021/ETH11409 respectively) and registered in the Australian New Zealand Clinical Trials Registry (respectively ACTRN12625000081415 and ACTRN12625000005459). Recruitment occurred between September 2022 and September 2024. Written informed consent was obtained from all participants and studies were conducted in accordance with the Declaration of Helsinki. Kidney donors had long‐term postoperative blood collected at 1 month, 3 months, and 1 year for kidney function.

### 2.2 Kidney functional reserve measurement

KFR was measured using one of three protocols:

- Protocol 1. A low‐protein diet (30‐40 g protein) for 24 h with overnight fasting for 8–12 h.
- Protocol 2. A normal diet preceding fasting overnight as in protocol 1.
- Protocol 3. A normal diet with fasting for 2–4 h prior to same‐day measurement.

Protocol 1 was used initially to maximize the difference between unstimulated and stimulated GFR, but evaluation of healthy volunteers by all three protocols detected no differences in magnitude of KFR by any functional marker (see Results). Subsequently, protocols 2 and 3 were used according to patient availability and preference. A single KFR test from each healthy volunteer was used in the final analysis. Kidney donors were offered follow‐up KFR tests at 1 and 3 months after surgery and measured postoperatively using the same protocol as preoperatively.

For KFR measurement, blood and urine were collected before and hourly for 5 h after ingestion of a 1.5 g/kg powdered whey protein “shake” (Iso Whey Zero, Biotech USA, in 300–600 mL water). Participants unable to tolerate the total volume completed protein loading with 10 g of nut‐derived protein and/or 25 g tuna protein.

Blood was collected in ethylenediaminetetraacetic acid and urine in polypropylene containers. Samples were centrifuged at 1700*g* at 4°C for 10 min within 1 h of collection. Plasma and urine supernatant samples were aliquoted and stored at −80°C until analysis.

Urine creatinine (uCr) (Thermo Fisher Scientific, Waltham, MA, USA, catalogue number 981896), pCr (Thermo Fisher Scientific, catalogue number 981896), and plasma CysC (Gentian Diagnostics, Moss, Norway, catalogue number B08179) were measured using a Konelab analyzer (Thermo Fisher Scientific™). Plasma PENK levels were assayed in blinded fashion using the sphingotest® penKid® immunoassay by SphingoTec GmbH (CLIA sphingotest® penKid®, Hennigsdorf, Germany, catalogue number 080‐02000). Clearance was normalized to body surface area using the Du Bois formula (Du Bois & Du Bois, 1989).

The coefficient of variation (CV) for CysC is 4%, for PENK 10%, for uCr 3.5%, for pCr 2%. These CVs were used to determine significant differences when comparing nadir concentrations of CysC, PENK, and pCr and peak uCr excretion times after protein.

### 2.3 Calculation of estimated glomerular filtration rates (eGFR)

CysC‐eGFR was calculated by Grubb's 2014 formula (CysC‐eGFR = 130×[(CysC)^−1.069^] × [(age)^−0.117^] −7) (Grubb et al., 2014), PENK‐eGFR by Beunders' 2023 formula (PENK‐eGFR = 10^[3.79–0.777×log(PENK) −0.324×log(Age)]^) (Beunders et al., 2023). Baseline eGFR was estimated by baseline pCr‐based CKD‐EPI 2021 formula, by pCr and CysC‐based CKD‐EPI 2021 formula (Inker et al., 2021) and by a creatinine‐independent estimate of GFR from the average of cystatin C‐ and PENK‐GFR (eGFR [CysC/PENK]). Baseline CKD was defined as eGFR <60 mL/min/1.73m^2^ (pCr‐based CKD‐EPI (Inker et al., 2021)).

Absolute KFR was the difference between peak and unstimulated (baseline) GFR (mL/min/1.73m^2^) and fractional KFR was absolute KFR divided by baseline GFR (× 100, %).

To facilitate method comparison, GFR at each timepoint was divided by baseline GFR: this normalized value was the fractional increase relative to unstimulated GFR.

### 2.4 Estimation of creatinine secretion

The contribution of tubular creatinine secretion to stimulated CrCl was estimated by two methods reliant on the creatinine‐independent estimates of GFR (eGFR [CysC/PENK]):

- by subtracting GFR from CrCl (i.e., CrCl – eGFR[CysC/PENK]) yielding the rate of tubular Cr secretion (with units of mL/min).
- by subtracting filtered Cr (pCr × average eGFR[CysC/PENK]) from Cr excreted in urine (UV Cr) at each timepoint: UV Cr – (pCr × eGFR[CysC/PENK]) yielding the mass of tubular Cr secretion (with units of μmol/min).

As tuna is rich in creatine, we hypothesized that patients receiving tuna would increase creatinine secretion and this would be revealed by a greater increase in CrCl but not CysC‐ or PENK‐eGFR.

### 2.5 Statistical analysis

Variables are shown as mean (±SD) or median (IQR) according to distribution. Categorical variables are shown as number (*n*) and percentage prevalence. Group comparisons were performed using the package Compare Groups of R studio (2023.06.0, which uses *t*‐test, Mann–Whitney, ANOVA, Kruskal–Wallis, Fisher, and chi‐squared depending on distribution and appropriateness).

Correlation between continuous variables was assessed by Pearson's or Spearman's correlation according to distribution. Bland–Altman graphs were designed to assess agreement between each pair of variables for baseline GFR and for absolute and fractional KFR, by plotting mean against difference between two variables. Mixed models with repeated measures were used for statistical analysis over time using JMP Software Pro 182024 (Cary, NC, USA). Models were adjusted by time, gender, age, baseline eGFR, BMI, and KFR protocol. Standard least squares graphs with confidence intervals were generated based on the mixed models.

Mixed models with repeated measures with compound symmetry unequal variances as the repeated structure were used to assess the intraclass correlation (ICC) of CysC‐eGFR, PENK‐eGFR, and CrCl to check consistency and homogeneity of the results with each biomarker. A higher ICC value indicates less variability within and between the groups.

Equivalence tests analysis was performed to assess the difference between successive values during KFR measurement. This used the median baseline value of each biomarker (CysC, PENK, or pCr) multiplied by the CV to determine whether this difference approached zero between two successive timepoints for each biomarker.

## 3 RESULTS

### 3.1 Participant characteristics

Sixty‐nine preoperative KFRs were obtained from 40 patients undergoing cardiac surgery and 22 healthy participants including 6 volunteers, 14 kidney donors, and 2 undergoing auto‐transplantation for nutcracker syndrome. Baseline characteristics are shown in Table 1. Four of 6 volunteers had 2 or 3 KFR measurements on different days to test diet and fasting protocols (13 KFRs). All 6 volunteers had KFR measured after protocol 2; 4 volunteers had KFR after protocol 1; 3 volunteers had KFR measured after protocol 3. (See Table S1 and 2 for additional details.) As the preparative protocol did not alter the results, final analysis included only KFRs performed using protocol 2 for all volunteers. Only 5 patients had CKD (eGFR <60 mL/min/1.73m^2^): all underwent cardiac surgery (median eGFR [IQR] 57 mL/min/1.73m^2^ [49;57.5]; [pCr range from 50 to 141 μmol/l]). Baseline GFR had good correlation and agreement when assessed by different methods, except for CrCl (Figures S1 and S2).

**TABLE 1:** Participant characteristics.

Baseline/Preoperative KFR tests	All	Healthy participants	Cardiac surgery	*p*‐value	*n*
	*n* = 62	*n* = 22	*n* = 40		
*Participant characteristics*					
Age (years), mean ± SD	61.8 ± 14.3	48.2 ± 11.7	69.2 ± 9.36	<0.001	62
Male gender, *n* (%)	44 (71.0)	10 (45.5)	34 (85)	0.003	62
BMI (kg/m^2^), median [IQR]	27.4 [24.1;30.4]	24.0 [22.9;28.8]	28.6 [25.5;31.0]	0.012	62
BSA (Du Bois) (m^2^), mean ± SD	1.98 ± 0.22	1.87 ± 0.23	2.04 ± 0.20	0.005	62
*Race*				0.005	62
White, *n* (%)	47 (75.8)	12 (54.5)	35 (87.5)		
Asian, *n* (%)	4 (6.45)	2 (9.1)	2 (5)		
Aboriginal/Torres strait islander, *n* (%)	1 (1.6)	0	1 (2.5)		
Middle East, *n* (%)	5 (8.1)	4 (18.2)	1 (2.5)		
Black, *n* (%)	1 (1.6)	0	1 (2.5)		
Latino, *n* (%)	2 (3.2)	2 (9.1)	0		
Other, *n* (%)	2 (3.2)	2 (9.1)	0		
Baseline pCr (μmol/L), median [IQR]	78.7 [65.4;86.3]	72.6 [61.2;80.0]	81.7 [74.4;100]	0.003	62
Baseline CysC (mg/L), median [IQR]	1.00 [0.88;1.25]	0.82 [0.72;0.91]	1.15 [1.01;1.37]	<0.001	62
Baseline PENK (pmol/L), median [IQR]	53.3 [42.9;74.2]	49.0 [42.4;64.5]	55.8 [45.2;77.8]	0.222	62
Baseline pCr‐eGFR (CKD‐EPI 2021) (mL/min/1.73m^2^), mean ± SD	90.6 ± 17.6	103 ± 11.7	83.8 ± 16.7	<0.001	62
Baseline [pCr/CysC]‐eGFR (CKD‐EPI 2021) (mL/min/1.73m^2^), mean ± SD	83.6 ± 22.9	104 ± 14.0	72.4 ± 18.7	<0.001	62
Baseline CysC‐eGFR (Grubb) (mL/min/1.73m^2^), mean ± SD	73.8 ± 24.5	97.5 ± 19.0	60.7 ± 16.0	<0.001	62
Baseline PENK‐eGFR (Beunders) (mL/min/1.73m^2^), mean ± SD	76.0 ± 23.6	87.5 ± 24.9	69.8 ± 20.6	0.004	62
Baseline CrCl (mL/min/1.73m^2^), mean ± SD	84.8 ± 35.7	100 ± 25.2	76.1 ± 38.1	0.005	61
Baseline average [CysC/PENK]‐eGFR (mL/min/1.73m^2^), mean ± SD	74.9 ± 21.9	92.5 ± 18.0	65.2 ± 17.5	<0.001	62
CKD, *n* (%)	5 (8.1)	0	5 (12.5)	0.151	62
COPD, *n* (%)	4 (8.7)	0	4 (10)	1	46
Diabetes mellitus, *n* (%)	18 (29)	0	18 (45)	0.001	62
Hypertension, *n* (%)	26 (41.9)	3 (13.6)	23 (57.5)	0.002	62
Cardiac ischaemia, *n* (%)	29 (46.8)	0	29 (72.5)	<0.001	62
Heart failure, *n* (%)	9 (14.5)	0	9 (22.5)	0.021	62

Abbreviations: BMI, body mass index; BSA, body surface area; CKD, eGFR <60 mL/min/1.73 m^2^; CKD‐EPI, chronic Kidney Disease Epidemiology Collaboration formula; COPD, chronic obstructive pulmonary disease; CrCl, creatinine clearance; CysC, cystatin C; eGFR, estimated glomerular filtration rate; IQR, interquartile range; pCr, plasma creatinine; PENK, proenkephalin‐A; SD, standard deviation.

Comparative results are shown for 3 different populations (Table 2): healthy participants (*n* = 22), cardiac surgery (*n* = 40), and all combined (*n* = 62). Of 14 kidney donors, 11 underwent 1‐month postoperative KFR and 10 also underwent 3‐month measurement.

**TABLE 2:** Fractional and absolute KFR.

KFR measurements	All	Healthy	Cardiac surgery	*p*‐value	*n*
	*n* = 62	*n* = 22	*n* = 40		
Fractional CysC‐based KFR (%), median [IQR]	10.5 [4.4; 16.2]	11.4 [7.0; 16.7]	9.4 [0.4; 13.6]	0.190	62
Absolute CysC‐based KFR (mL/min/1.73m^2^), median [IQR]	7.0 [2.5; 11.7]	11.6 [6.6; 15.1]	5.0 [0.3; 8.7]	0.002	62
Fractional PENK‐based KFR (%), median [IQR]	44.2 [35.5; 58.0]	45.8 [36.0; 59.2]	43.6 [33.7; 58.6]	0.546	62
Absolute PENK‐based KFR (mL/min/1.73m^2^), median [IQR]	30.5 [22.0; 52.0]	33.8 [26.6; 59.0]	28.5 [19.9; 40.6]	0.046	62
Fractional CrCl‐based KFR (%), median [IQR]	30.0 [8.4;68.9]	28.7 [9.9; 46.0]	31.8 [−0.3; 95.5]	0.476	60
Absolute CrCl‐based KFR (mL/min/1.73m^2^), median [IQR]	23.7 [8.9; 43.6]	23.7 [11.9; 43.6]	23.4 [−0.3; 43.9]	0.602	60

Abbreviations: CrCl, creatinine clearance; CysC, cystatin C; IQR: interquartile range; KFR, kidney functional reserve; PENK, proenkephalin‐A.

### 3.2 Diet and fasting preparatory protocols had no influence on KFR results

Mixed model analysis of repeated measures adjusted by timepoints, gender, age, baseline eGFR, BMI, KFR protocol, and the interaction between KFR protocol and timepoints showed no differences among the preparatory protocols (Figure S3). There was no difference in KFR (all calculation methods) between the different preparative protocols (Table S2). There was no difference in tubular secretion rate and absolute creatinine secretion in the 13 KFRs of the 6 healthy volunteers between the preparative protocols (Figure S4).

### 3.3 Low‐protein diet and long fasting time associated with adverse events

The main reported side effect of KFR measurement was nausea, occurring in 19.4% of participants (Table S1). Nausea was more common after protocol 1 (50% of patients, compared to 13% in the other two preparatory protocols, *p* = 0.03). Diarrhea was similar in the three protocols (6.45% incidence in combined cohort). No other adverse events were reported.

### 3.4 PENK KFR demonstrated greater dynamic range and homogeneity

**FIGURE 1:**
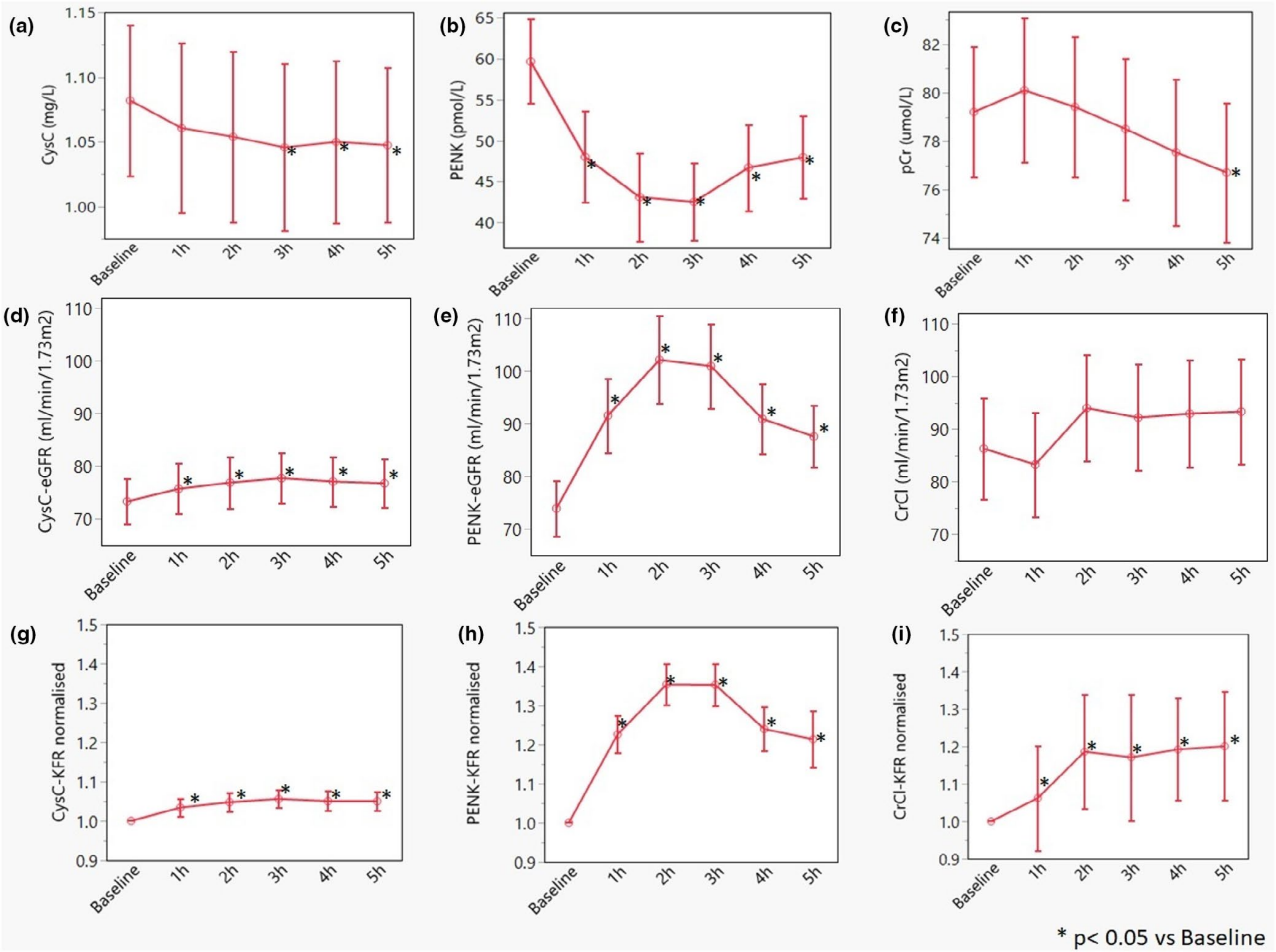
Plasma Biomarkers and Kidney functional reserve in total cohort (1 test per participant; *n* = 62). (a) Plasma cystatin C, in mg/L; (b) Plasma PENK, in pmol/L; (c) Plasma creatinine, in μmol/L; (d) CysC‐eGFR (mL/min/1.73m^2^); (e) PENK‐eGFR (mL/min/1.73m^2^); (f) CrCl, in mL/min/1.73m^2^; (g) Fractional CysC‐eGFR (normalized to baseline); (h) Fractional PENK‐eGFR (normalized to baseline); (i) Fractional CrCl (normalized to baseline). Mixed models were adjusted by time, gender, age, baseline eGFR, BMI, KFR protocol. Significance: *p < 0.05 versus baseline. Exact *p*‐values versus Baseline: (a) CysC (mg/L): T1: *p* = 0.2169; T2: *p* = 0.0503; T3: *p* = 0.0054; T4: *p* = 0.0328; T5: *p* = 0.0129. (b) PENK (pmol/L): T1: *p* < 0.0001; T2: *p* < 0.0001; T3: *p* < 0.0001; T4: *p* < 0.0001; T5: *p* < 0.0001. (c) pCr (μmol/L): T1: *p* = 0.3763; T2: *p* = 0.9531; T3: *p* = 0.7565; T4: *p* = 0.2378; T5: *p* = 0.0256. (d) CysC‐eGFR (mL/min/1.73 m^2^): T1: *p* = 0.0110; T2: *p* = 0.0014; T3: *p* = 0.0002; T4: *p* = 0.0028; T5: *p* = 0.0089. (e) PENK‐eGFR (mL/min/1.73 m^2^): T1: *p* < 0.0001; T2: *p* < 0.0001; T3: *p* < 0.0001; T4: *p* < 0.0001; T5: *p* < 0.0001. (f) CrCl (mL/min/1.73 m^2^): T1: *p* = 0.9292; T2: *p* = 0.2556; T3: *p* = 0.5270; T4: *p* = 0.4087; T5: *p* = 0.3582. (g) CysC‐KFR normalized: T1: *p* = 0.0028; T2: *p* = 0.0001; T3: *p* < 0.0001; T4: *p* = 0.0001; T5: *p* < 0.0001. (h) PENK‐KFR normalized: T1: *p* < 0.0001; T2: *p* < 0.0001; T3: *p* < 0.0001; T4: *p* < 0.0001; T5: *p* < 0.0001. (i) CrCl‐KFR normalized: T1: *p* = 0.4028; T2: *p* = 0.0189; T3: *p* = 0.0498; T4: *p* = 0.0066; T5: *p* = 0.0076.

**FIGURE 2:**
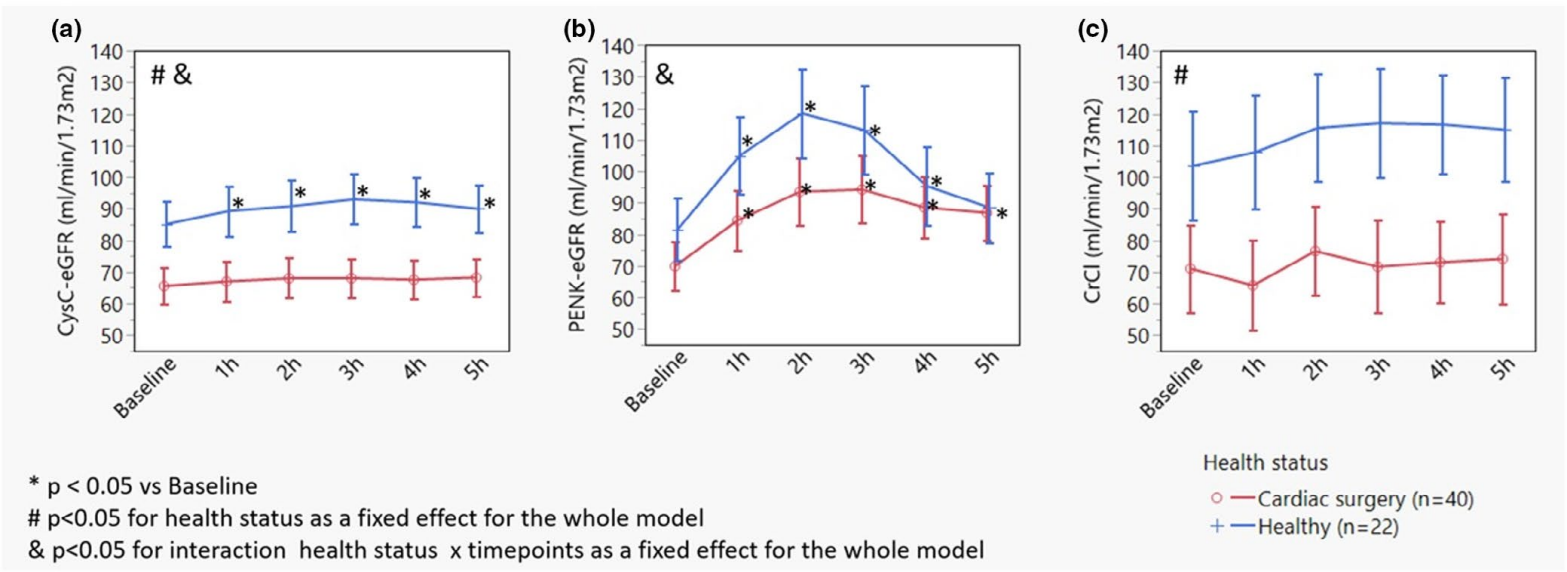
Kidney functional reserve, according to health status (healthy participants vs. cardiac surgery patients). (a) CysC‐eGFR, in mL/min/1.73 m^2^; (b) PENK‐eGFR, in mL/min/1.73 m^2^; (c) CrCl, in mL/min/1.73 m^2^. Timepoints: baseline (before protein) and 1, 2, 3, 4, and 5 h after protein. Red: “cardiac surgery”; Blue: healthy participants (volunteers, kidney donors, and people undergoing kidney auto‐transplantation). Values represent standard least mean squares and confidence intervals. Mixed models were adjusted by time, gender, age, baseline eGFR, BMI, KFR protocol, health status, and the interaction between health status and timepoints. Significance: *p < 0.05 vs. Baseline; #*p* < 0.05 for health status as a fixed effect for the whole model; & *p* < 0.05 for interaction health status × timepoints as a fixed effect for the whole model. Exact *p*‐values versus baseline: (a) CysC‐eGFR (mL/min/1.73 m^2^), by health status: Healthy: T1: *p* = 0.0134; T2: *p* = 0.0005; T3: *p* < 0.0005; T4: *p* < 0.0005; T5: *p* = 0.0129/Cardiac surgery: T1: *p* = 0.5552; T2: *p* = 0.0901; T3: *p* = 0.0695; T4: *p* = 0.3355; T5: *p* = 0.1053. Health status as a fixed effect: *p* < 0.0001; interaction health status × timepoints as a fixed effect: *p* = 0.0010. (b) PENK‐eGFR (mL/min/1.73 m^2^), by health status: Healthy: T1: *p* < 0.0005; T2: *p* < 0.0005; T3: *p* < 0.0005; T4: *p* = 0.0035; T5: *p* = 0.2594/Cardiac surgery: T1: *p* < 0.0005; T2: *p* < 0.0005; T3: *p* < 0.0005; T4: *p* < 0.0005; T5: *p* < 0.0005. Health status as a fixed effect: *p* = 0.0787; interaction health status × timepoints as a fixed effect: *p* = 0.0158. (c) CrCl (mL/min/1.73 m^2^), by health status: Healthy: T1: *p* = 0.9962; T2: *p* = 0.5204; T3: *p* = 0.3816; T4: *p* = 0.4099; T5: *p* = 0.6263/Cardiac surgery: T1: *p* = 0.9309; T2: *p* = 0.9431; T3: *p* = 1.0000; T4: *p* = 1.0000; T5: *p* = 0.9995. Health status as a fixed effect: *p* = 0.0003; interaction health status × timepoints as a fixed effect: *p* = 0.7641.

Protein ingestion reduced CysC, PENK, and pCr (Figure 1a–c), analogous to stimulation of CysC‐eGFR, PENK‐eGFR, and CrCl (Figure 1d–f). The increase in GFR was best seen with PENK‐eGFR for both healthy participants and cardiac surgery patients. The increase in CysC‐eGFR was not significant in patients undergoing cardiac surgery but was significant in the healthy cohort (Figure 2).

The eGFR normalized to baseline and the fractional KFR increases highlighted the increase in dynamic range of eGFR, which was greatest for PENK (Figure 1g–i, Table 2). The median [IQR] fractional KFR for PENK was 44.2% [35.5; 58.0]; for CysC 10.5% [4.4; 16.2], and for CrCl 30.0% [8.4; 68.9] (*p* < 0.0001).

Individual results were more homogeneous with CysC‐ and PENK‐eGFR than CrCl as demonstrated by the higher intraclass correlation of measurements over time: 0.98 for CysC‐eGFR, 0.86 for PENK‐eGFR and 0.63 for CrCl. See also Figure S5.

### 3.5 Only CysC‐ and PENK‐KFR detected differences between healthy participants and cardiac surgery patients

Median absolute KFR estimates differed significantly between healthy and cardiac surgery groups when assessed using CysC or PENK (Table 2, Figure 2). Patients undergoing cardiac surgery had lower absolute CysC and PENK KFRs compared to healthy participants. There was no difference when assessed by absolute CrCl‐KFR. Differences in fractional KFR were not present between the groups regardless of biomarker method.

Better correlation and agreement were seen for CysC‐ and PENK‐KFR, while CrCl‐KFR seemed to provide different information from CysC‐ and PENK‐KFR (Figures S6–, S8).

### 3.6 CysC and PENK GFR defined KFR earlier than creatinine‐based measurements

**FIGURE 3:**
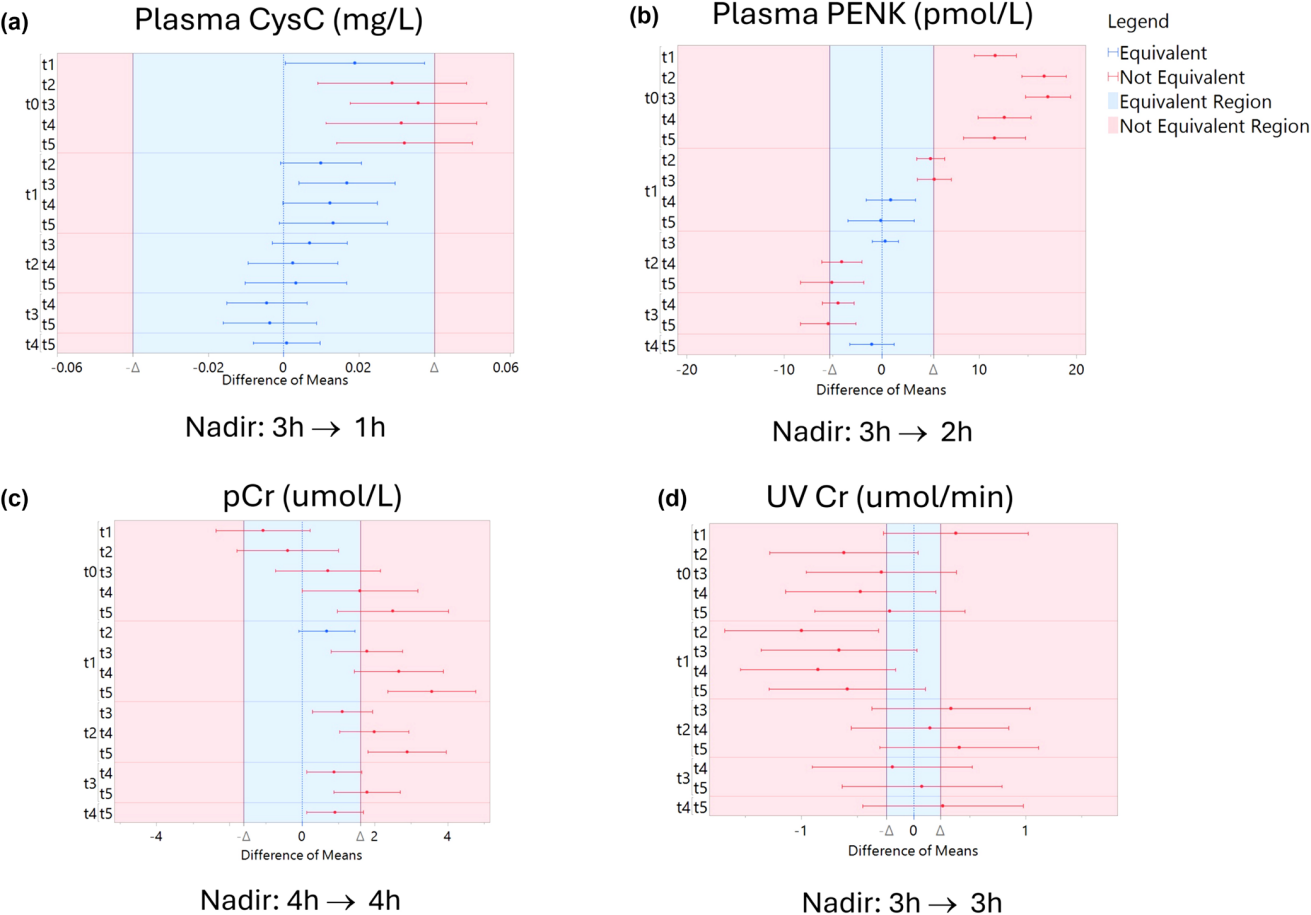
Forrest plots for equivalence based on biomarker CVs to determine two statistically different values (*n* = 62). Differences between the values of: (a) plasma CysC (mg/L); (b) plasma PENK (pmol/L); (c) pCr (μmol/L); and (d) UV Cr (creatinine urine excretion, in μmol/min) for each 2 timepoints. The *x*‐axis shows the difference of means. The y‐axis shows different timepoints; t0 represents baseline, t1–t5 represent 1–5 h after the protein. The median time to reach the nadir of CysC would be 3 h, but equivalence tests show t3 = t2 = t1, so nadir was reached in 1 h for CysC. The median time to reach the nadir of PENK would be 3 h, but equivalence tests show t3 = t2, so nadir was reached in 2 h for PENK. The median time to reach the nadir of pCr would be 4 h, and equivalence tests show t4 is different from t3, so nadir was reached in 4 h for pCr. The median time to reach the peak of creatinine excretion (UV Cr) would be 3 h, and equivalence tests show t3 is different from t2, so peak was reached in 3 h for UV Cr.

Equivalence test analysis using laboratory CVs to discriminate detectable differences between biomarker concentrations showed nadir CysC occurred 1 h (h) after protein: while the observed nadir was at 3 h, values at 3 h were equivalent to values at 2 h and 1 h. For PENK, the nadir occurred at 2 h (values at 3 h were equivalent to values at 2 h). For creatinine‐based measurements there was no equivalence among values and nadir pCr occurred at 4 h, with peak Cr excretion at 3 h (Figure 3). In healthy participants, the nadir CysC, PENK and pCr occurred respectively within 2, 2, and 4 h considering the laboratory CVs, and the creatinine excretion (UV Cr) peak happened within 3 h. In cardiac surgery participants, nadir CysC, PENK and pCr were respectively at 1, 2, and 3 h with UV Cr peak at 3 h.

### 3.7 KFR and baseline kidney function provided different information

**FIGURE 4:**
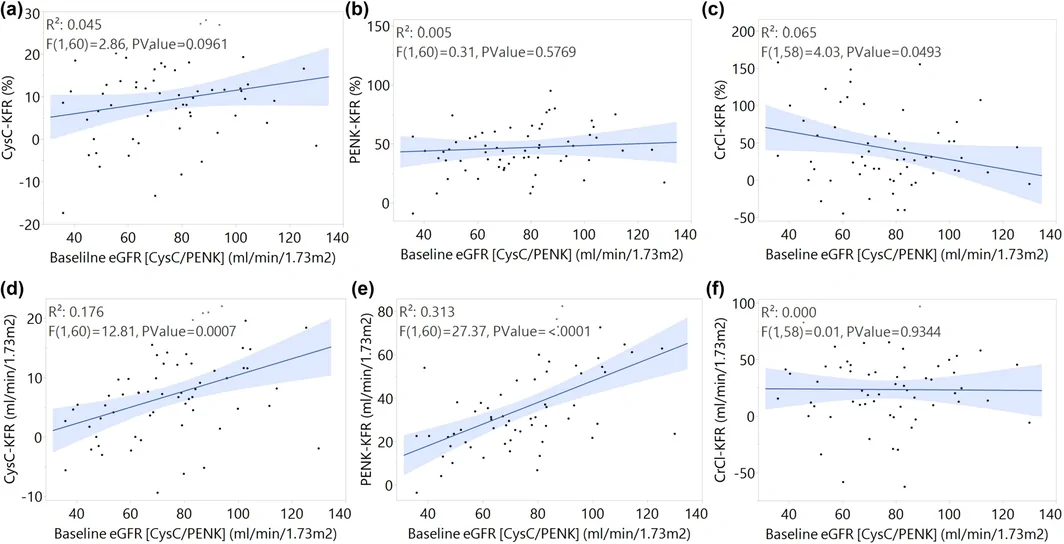
Correlation between KFR by different biomarkers and baseline kidney function as determined by eGFR [CysC/PENK]). (a–c) Fractional KFR; D‐F: Absolute KFR. (a) CysC‐KFR (%) vs. baseline eGFR [CysC/PENK]; (b) PENK‐KFR (%) vs. baseline eGFR [CysC/PENK]; (c) CrCl‐KFR (%) vs. baseline eGFR [CysC/PENK]; (d) CysC‐KFR (in mL/min/1.73 m^2^) vs. baseline eGFR [CysC/PENK]; (e) PENK‐KFR (in mL/min/1.73 m^2^) vs. baseline eGFR [CysC/PENK]; (f) CrCl‐KFR (in mL/min/1.73 m^2^) vs. baseline eGFR [CysC/PENK].

Absolute CysC‐ and PENK KFR correlated positively with baseline eGFR, albeit modestly (Figure 4, Figure S9). Neither fractional CysC‐ nor PENK KFR (%) correlated with baseline eGFR by [CysC/PENK] or pCr‐eGFR (CKD‐EPI 2021). Fractional CrCl KFR correlated negatively and modestly with baseline eGFR (Figure 4, Figure S9). Similarly, tubular creatinine secretion correlated minimally and negatively with baseline function (Figure S10).

### 3.8 Stimulated CrCl represented tubular secretion increases in addition to glomerular creatinine filtration

Mixed model analysis assessing the impact of using tuna as an additional source of protein showed no impact of tuna ingestion on the biomarker profiles for CysC or PENK, but significant differences for pCr and urine creatinine excretion (UV Cr) (Figure S11A–D). Despite the patients receiving tuna (*n* = 7) having slightly higher levels of CysC, PENK, and pCr, reflecting slightly lower eGFRs for CysC and PENK, urine creatinine excretion in these patients was significantly higher so that CrCl also increased (Figure S11) with similar profiles for UV Cr and CrCl (Figure S11D,G). In most, the rate and mass of tubular Cr secretion decreased slightly after whey protein loading (Figure S12). However, participants who received tuna had increased tubular Cr secretion approximating the increase in CrCl (Figures S11 and S12).

### 3.9 Effect of kidney donation on KFR

One of 14 kidney donors had hypertension; none had diabetes, coronary disease, or heart failure. Donor median [IQR] body mass index (BMI) was 27.8 [22.9; 30.8] kg/m^2^; 4 donors had BMI higher than 30 kg/m^2^. Donor median age [IQR] was 55 [49.7; 61.5] years (range: 36 to 64 years).

**FIGURE 5:**
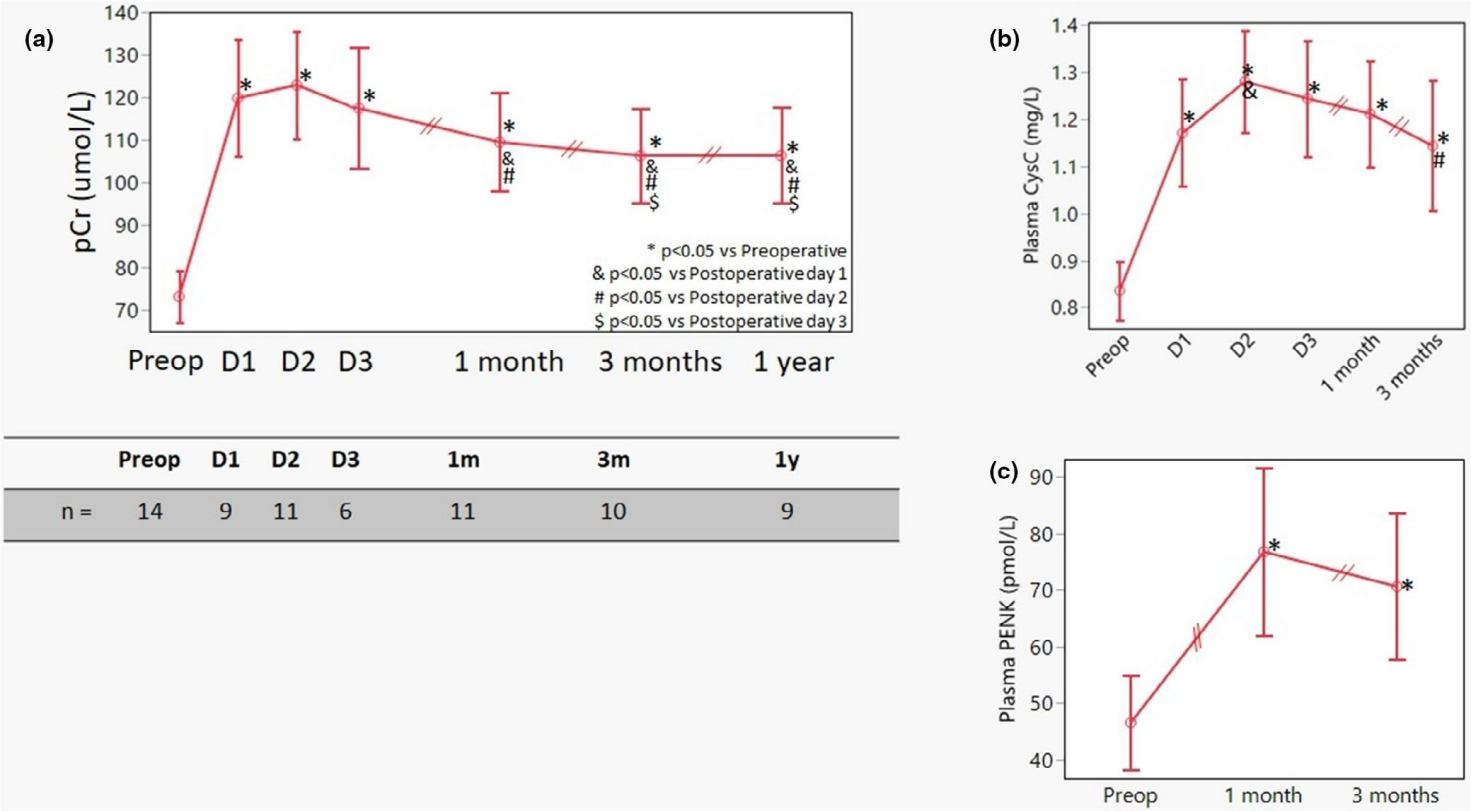
Kidney function biomarkers before and after kidney donation. (a) Plasma creatinine (μmol/L), *x*‐axis time: Preoperative, and 1, 2, and 3 days, 1 month, 3 months, and 1 year after surgery; (b) plasma cystatin C (mg/L), *x*‐axis time: Preoperative, and 1, 2, and 3 days, 1 month and 3 months after surgery; (c) plasma PENK (pmol/L), *x*‐axis time: Preoperative and 1 month and 3 months after surgery. Values depict standard least mean squares and confidence intervals. The number of patients with plasma samples collected at each timepoint is shown below Panel A. Significance levels shown in panels B and C are as in Panel A. Exact *p*‐values versus Preoperative: (a) pCr (μmol/L): D1: *p* < 0.0001; D2: *p* < 0.0001; D3: *p* < 0.0001; 1 month: *p* < 0.0001; 3 months: *p* < 0.0001; 1 year: *p* < 0.0001. (b) Plasma CysC (mg/L): D1: *p* < 0.0001; D2: *p* < 0.0001; D3: *p* < 0.0001; 1 month: *p* < 0.0001; 3 months: *p* < 0.0001. (c) Plasma PENK (pmol/L): 1 month: *p* < 0.0001; 3 months: *p* < 0.0001. Exact *p*‐values versus Postoperative D1: (a) pCr (μmol/L): D2: *p* = 0.8117; D3: *p* = 0.9677; 1 month: *p* = 0.0155; 3 months: *p* = 0.0020; 1 year: *p* = 0.0027. (b) Plasma CysC (mg/L): D2: *p* = 0.0171; D3: *p* = 0.3203; 1 month: *p* = 0.6508; 3 months: *p* = 0.9483. Exact *p*‐values versus Postoperative D2: (a) pCr (μmol/L): D3: *p* = 0.5313; 1 month: *p* = 0.0004; 3 months: *p* < 0.0001;1 year: *p* < 0.0001. (b) Plasma CysC (mg/L): D3: *p* = 0.8666; 1 month: *p* = 0.1736; 3 months: *p* = 0.0102. Exact *p*‐values versus Postoperative D3: (a) pCr (μmol/L): 1 month: *p* = 0.1409; 3 months: *p* = 0.0276; 1 year: *p* = 0.0300. (b) Plasma CysC (mg/L): 1 month: *p* = 0.8589; 3 months: *p* = 0.1311. Exact *p*‐values versus 1‐month postoperative: (a) pCr (μmol/L): 3 months: *p* = 0.7199; 1 year: *p* = 0.7755. (b) Plasma CysC (mg/L): 3 months: *p* = 0.3116. (c) Plasma PENK (pmol/L): 3 months: *p* = 0.1112. Exact *p*‐values versus 3‐months postoperative: (a) pCr (μmol/L): 1 year: *p* = 1.0000.

Long‐term donor follow‐up showed pCr increased immediately after nephrectomy, then decreased over 1 month. At 1 month after donation pCr values approximated those 1 year after donation (Figure 5a). Median preoperative eGFR [IQR] for donors was 97 [91;106] mL/min/1.73m^2^, then 59 [51;67] at 1 month, 59 [55;74] at 3 months and 57 [52;65] at 1 year. Similar findings were observed with plasma CysC and PENK levels up to 3 months after donation (Figure 5b,c). No PENK or CysC values were available after 3 months.

**FIGURE 6:**
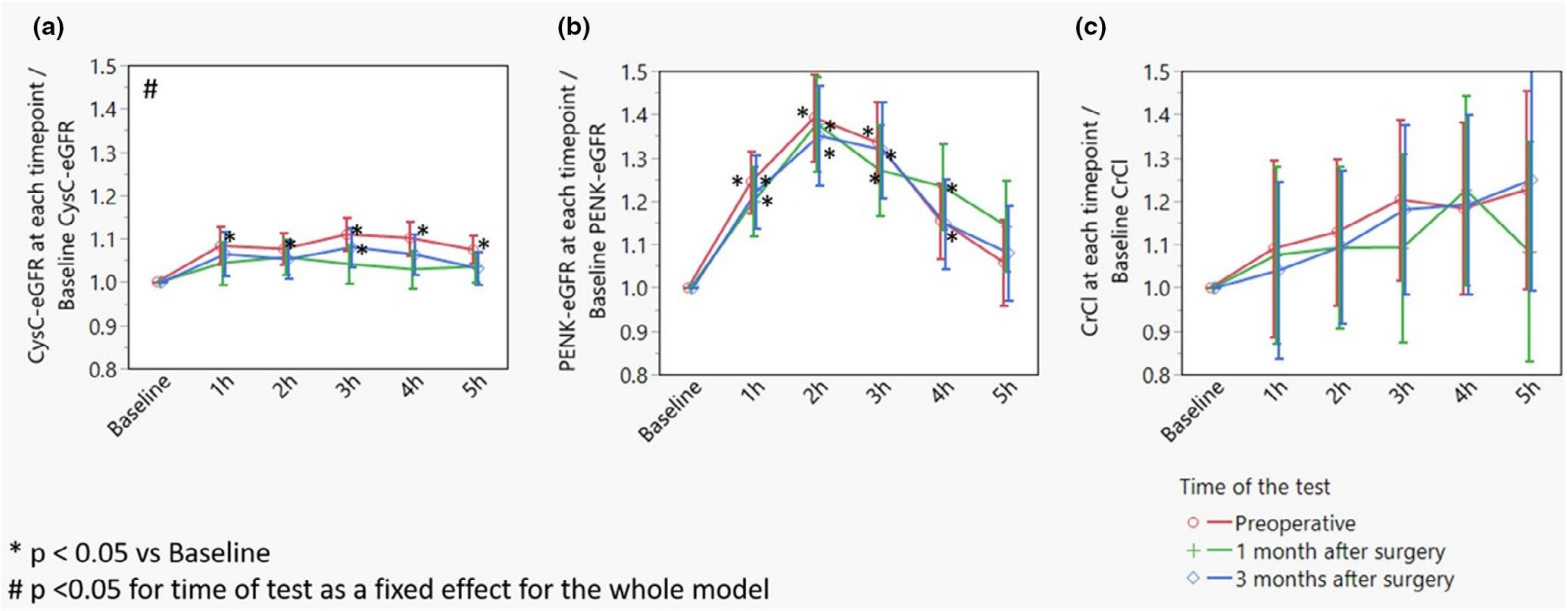
Fractional Kidney functional reserve before and after kidney donation. (a) Fractional CysC‐eGFR; (b) Fractional PENK‐eGFR; (c) Fractional CrCl. *X*‐axis time, before (baseline) and 1, 2, 3, 4, and 5 h after oral protein. Red: preoperative (*n* = 14); green: 1‐month postoperative (*n* = 11); blue: 3‐month postoperative (*n* = 10). Values depict standard least mean squares and confidence intervals. Mixed models were adjusted by timepoint, gender, age, time of test, and the interaction between time of test and timepoint). Significance: *p < 0.05 versus baseline; #*p* < 0.05 for time of the test as a fixed effect for the whole model. Exact *p*‐values versus Baseline: (A) CysC‐eGFR at each timepoint/Baseline CysC‐eGFR: Preoperative: T1: *p* = 0.0023; T2: *p* = 0.0009; T3: *p* < 0.0001; T4: *p* < 0.0001; T5: *p* = 0.0001/1 month after surgery: T1: *p* = 0.6554; T2: *p* = 0.0697; T3: *p* = 0.5679; T4: *p* = 0.9494; T5: *p* = 0.4771/3 months after surgery: T1: *p* = 0.2047; T2: *p* = 0.2176; T3: *p* = 0.0094; T4: *p* = 0.1196; T5: *p* = 0.7741. Time of the test as a fixed effect for the whole model: *p* = 0.0006; interaction time of the test × timepoints as a fixed effect for the whole model: *p* = 0.0669. (b) PENK‐eGFR at each timepoint/Baseline PENK‐eGFR: Preoperative: T1: *p* < 0.0001; T2: *p* < 0.0001; T3: *p* < 0.0001; T4: *p* = 0.0095; T5: *p* = 0.9816/1 month after surgery: T1: *p* < 0.0001; T2: *p* < 0.0001; T3: *p* < 0.0001; T4: *p* = 0.0002; T5: *p* = 0.1116/3 months after surgery: T1: *p* < 0.0001; T2: *p* < 0.0001; T3: *p* < 0.0001; T4: *p* = 0.0969; T5: *p* = 0.9355. Time of the test as a fixed effect for the whole model: *P* = 0.7642; interaction time of the test × timepoints as a fixed effect for the whole model: *p* = 0.8701. (c) CrCl at each timepoint/Baseline CrCl: T1: Preoperative: T1: *p* = 0.9990; T2: *p* = 0.8567; T3: *p* = 0.3417; T4: *p* = 0.6187; T5: *p* = 0.5177/1 month after surgery: T1: *p* = 1.0000; T2: *p* = 0.9983; T3: *p* = 0.9998; T4: *p* = 0.5018; T5: *p* = 1.000/3 months after surgery: T1: *p* = 1.000; T2: *p* = 0.9979; T3: *p* = 0.7252; T4: *p* = 0.7409; T5: *p* = 0.6716. Time of the test as a fixed effect for the whole model: *p* = 0.6657; interaction time of the test × timepoints as a fixed effect for the whole model: *p* = 0.9947.

Despite decreased baseline GFR after nephrectomy, KFR remained detectable at 1 and 3 months, as most easily observed with PENK (Figure 6). Median fractional PENK‐KFR [IQR] at 1 and 3 months was 38% [29; 59] and 40% [30; 50]. Thus, although absolute KFR decreased postoperatively, fractional KFR remained intact after kidney donation (Figure S13).

## 4 DISCUSSION

This is the first study to examine the use of PENK to measure KFR. We hypothesized that using PENK could shorten the duration of KFR assessment. The results suggest that PENK was an excellent functional biomarker for KFR determination and allowed more rapid assessment of KFR compared to creatinine‐based methods. Overall, the nadir PENK after protein occurred within 2 h and nadir CysC within 1 h after protein ingestion. In healthy subjects, the PENK and CysC nadir occurred at the same time and was thus comparable to a 2 h oral glucose tolerance test. PENK provided a greater dynamic range than CysC or pCr‐based methods, which facilitated trough PENK vs. peak‐eGFR discrimination and the PENK‐based KFR results were homogeneous among participants. PENK‐KFR and CysC‐KFR showed similar temporal behavior patterns, which differed from creatinine clearance‐KFR, especially in response to meat or tuna‐derived protein and differentiated between healthy subjects and polymorbid patients.

The reasons for PENK's greater dynamic range after protein loading remain unclear. Interestingly, we observed occasional negative KFR responses with PENK, similar to those we and others have previously observed with CrCl and Cystatin‐C KFR (Christiadi et al., 2022; Sharma et al., 2016). PENK is a stable 4.5 kDa 41 amino acid‐long peptide that is freely filtered by the kidneys after generation during metabolism from precursor proteins to mature endogenous opioid enkephalins (Donato et al., 2018). Plasma PENK concentration correlates strongly with GFR (Beunders et al., 2020; Khorashadi et al., 2020). In AKI, PENK levels increase before pCr (ESICM LIVES 2019; Hollinger et al., 2018; Matsue et al., 2017). In rats, high fat intake is associated with increased enkephalin expression, while a calorie‐dense high‐protein diet can reduce enkephalin expression (Genders et al., 2020; Kelley et al., 2005), potentially through increased kidney filtration. Proposed mechanisms for the increased GFR after oral protein include increased amino acid filtration, higher proximal tubular sodium absorption with decreased delivery of sodium and chloride to the macula densa, thereby inhibiting tubuloglomerular feedback and inducing afferent arteriolar vasodilation and increased blood flow. Amino acids stimulate nitric oxide (NO) and local prostaglandin (PG) release, further inhibiting tubuloglomerular feedback (Jufar et al., 2020). Interaction with glucagon allows GFR increase after a protein meal with a permissive role for modulation by NO and PG (Jufar et al., 2020; Premen, 1989; Premen et al., 1990; Wada et al., 1991). The reported reduction in post‐operative low‐perfusion AKI after cardiac surgery has been attributed to recruitment of resting nephrons by amino acid stimulation (Landoni et al., 2024).

Despite the predominant use of CrCl in previous KFR studies (Husain‐Syed et al., 2018; Palsson & Waikar, 2018), the latency in pCr change, the presence of tubular creatinine secretion (Khorashadi et al., 2020) and the difficulty in obtaining reliable hourly measurements in uncatheterised subjects have limited widespread clinical KFR assessment. Indeed, even baseline unstimulated CrCl differed significantly from baseline GFR assessed by the other methods in this study, suggesting CrCl measures something else in addition to GFR. It was clear in this study that CrCl‐based KFR was driven by increased tubular creatinine secretion rather than GFR change after tuna protein loading, consistent with the known increase in creatinine secretion stimulated by creatinine loading (Herrera & Rodríguez‐Iturbe, 1998).

Fractional CrCl‐KFR and tubular creatinine secretion were greater at lower baseline eGFR. Patients with CKD have increased tubular creatinine secretion relative to total CrCl, accounting for up to 50% of CrCl in advanced CKD (Ellam, 2011). Proposed mechanisms include upregulation of creatinine and organic ion secretion transporters in CKD in response to increases in uraemic toxins (Ellam, 2011; Toyohara et al., 2009). There might also be disproportionate loss of glomerular relative to tubular secretory function. However, even tubular creatinine secretion can be reduced in severe CKD (Herrera & Rodríguez‐Iturbe, 1998). A previous study showed that patients with plasma creatinine ranging from 212 to 486 μmol/l were unable to increase creatinine secretion after protein (Herrera & Rodríguez‐Iturbe, 1998). CKD patients in the present study had less severe kidney disease, with pCr ranging from 50 to 141 μmol/l, a range where increased tubular Cr secretion could occur despite GFR reduction. The methods of determining tubular creatinine secretion in this study, combining CrCl with PENK‐GFR and/or CysC‐GFR, could be utilized to measure creatinine secretion in future studies to determine if variations in tubular function are useful in predicting CKD outcomes. Previous studies have already shown that the average of eGFR by two different biomarkers, similar to calculations in this study, might be useful for estimating kidney function (Morizawa et al., 2020).

When measured by CysC or PENK, absolute but not fractional KFR was positively correlated with baseline kidney function. Both CysC‐ and PENK‐ absolute KFRs differentiated healthy participants from patients with comorbidities undergoing cardiac surgery. This is consistent with the correlations between baseline eGFR and the absolute KFR when measured by either CysC or PENK. Among these patients, the differentiation appeared somewhat more significant when measured using CysC, although some overlap remains between groups with both methods. However, the more critical observation is that these differences were absent when KFR and baseline eGFR were measured using CrCl. In the cardiac surgery population, a low KFR might be a consequence of multiple factors, including aging, CKD, cardiorenal interaction, or other confounding factors. For differentiation between healthy people and patients with multiple comorbidities, CysC apparently had some advantage, as numbers seemed to be more expressive in differentiating those populations for CysC‐ than for PENK‐absolute KFR. This result confirms that KFR needs to be measured directly, as the extent of increase in GFR after protein is not predictable from baseline eGFR, particularly for creatinine clearance. Absolute KFR may enhance interpretation of sCKD severity, as KFR incorporates baseline kidney function.

We hypothesized that KFR might be recruited after kidney donation as part of compensatory hypertrophy or hyperfiltration. Absolute KFR decreased after kidney donation, reflecting decreased baseline GFR. However, this study revealed that fractional KFR was preserved after kidney donation. This suggests that increases in GFR after nephrectomy in kidney donors occur early and independently of KFR recruitment.

Study limitations include modest participant numbers in a single centre and absent independent measures of tubular creatinine secretion and GFR. Comparisons with cystatin C GFR and KFR provide strong validation since CysC‐KFR has been previously validated against Tm‐DTPA GFR (Christiadi et al., 2022). Another study limitation was the absence of a direct measurement of GFR as a comparator, because of the limited time most patients had to participate in the study prior to surgery and the near impossibility of performing simultaneous GFR measurements by alternative methods in a ‘real‐time’ manner. However, given that iothalamate, DTPA and other current direct measures of GFR are also delayed approximations averaged over hours, no true direct comparisons with real‐time GFR are yet possible. A future solution to facilitate interpretation of GFR changes after protein loading could be estimation of GFR using relmapyrazin (Dorshow et al., 2024). The absence of comorbidities in the kidney donors limits conclusions regarding KFR in expanded criteria donors. In addition, the evidence in patients with CKD is limited in our data, as only five patients with mild CKD were included, limiting extrapolation regarding PENK‐KFR in CKD risk stratification, disease progression, and clinical management. Further evaluation including assessment of predictive performance with respect to relevant clinical outcomes remains necessary to establish the medical utility of PENK‐KFR.

In summary, PENK facilitated rapid estimations of GFR reserve, with reproducible homogeneous results similarly not influenced by tubular secretion but with a greater dynamic range than CysC. The short time to peak GFR, especially where baseline eGFR was normal, absent need for urine collection, minimal fasting requirement, plasma collection for only 2 or 3‐h and absence of endogenous or exogenous chemical interferences should facilitate clinical use of PENK‐KFR as a kidney stress test. KFR measurements using PENK‐ and CysC provided similar information regarding glomerular function but differed from CrCl‐KFR. CrCl‐KFR may retain utility as a measure of tubular functional reserve.

## AUTHOR CONTRIBUTIONS

**Camila Eleuterio Rodrigues:** Study design data curation; formal analysis; funding acquisition; investigation; methodology; project administration; resources; software; visualization. **Amy Y. M. Au:** Methodology; resources; visualization. **Daniel Rebello:** Methodology. **Maria Maawad:** Methodology. **Florian Uhle:** Funding acquisition; methodology; validation; visualization. **Jonathan Erlich:** Study design; formal analysis; investigation; methodology; resources; software; supervision; validation; visualization. **Zoltan H. Endre:** Conceptualization; study design, data curation; funding acquisition; investigation; methodology; project administration; resources; software; supervision; validation; visualization.

## FUNDING INFORMATION

Camila Eleuterio Rodrigues was supported by Fundação de Amparo à Pesquisa do Estado de São Paulo (FAPESP), Brazil, grant number 2019/19631‐7. Camila Eleuterio Rodrigues and Zoltán H. Endre received support from the Prince of Wales Hospital and Lewis Foundations. Plasma PENK levels were provided by SphingoTec GmbH (Hennigsdorf, Germany). This study was supported by funding to the Australian Kidney Biomarker Reference Laboratory and funding from the Prince of Wales Hospital Foundation, Sydney, Australia.

## CONFLICT OF INTEREST STATEMENT

Plasma PENK levels were assayed in blinded fashion using the sphingotest® penKid® immunoassay by SphingoTec GmbH (Hennigsdorf, Germany). Florian Uhle is a current employee of SphingoTec GmbH and holder of stock options from SphingoTec GmbH. Zoltan Endre and the Prince of Wales Hospital have received payment for conceptualization of PenKid use to measure KFR from SphingoTec GmbH (Henningsdorf, Germany).

## DISCLAIMERS

The content is solely the responsibility of the authors and does not necessarily represent the official views of SphingoTec GmbH (Hennigsdorf, Germany).

## Supporting information


**Figure S1.** Correlation between baseline unstimulated glomerular filtration rate measured by different methods. (A) Cystatin C‐based eGFR by Grubb formula versus CKD‐EPI formula‐based eGFR using both plasma creatinine and cystatin C; (B) Proenkephalin‐A‐based eGFR by Beunders formula versus CKD‐EPI formula‐based eGFR using both plasma creatinine and cystatin C; (C) Proenkephalin‐A‐based eGFR by Beunders formula versus cystatin C‐based eGFR by Grubb formula; (D) Creatinine clearance versus CKD‐EPI formula‐based eGFR using both plasma creatinine and cystatin C; (E) Creatinine clearance versus cystatin C‐based eGFR by Grubb formula; (F) Creatinine clearance versus proenkephalin‐A‐based eGFR by Beunders formula; (G) CKD‐EPI formula‐based eGFR using plasma creatinine only versus CKD‐EPI formula‐based eGFR using both plasma creatinine and cystatin C; (H) CKD‐EPI formula‐based eGFR using plasma creatinine only versus cystatin C‐based eGFR by Grubb formula; (I) CKD‐EPI formula‐based eGFR using plasma creatinine only versus proenkephalin‐A‐based eGFR by Beunders formula; (J) CKD‐EPI formula‐based eGFR using plasma creatinine only versus creatinine clearance; (K) average of cystatin C‐ and proenkephalin‐A GFR versus CKD‐EPI formula‐based eGFR using both plasma creatinine and cystatin C; (L) average of cystatin C‐ and proenkephalin‐A GFR versus cystatin C‐based eGFR by Grubb formula; (M) average of cystatin C‐ and proenkephalin‐A GFR versus proenkephalin‐A‐based eGFR by Beunders formula; (N) average of cystatin C‐ and proenkephalin‐A GFR versus creatinine clearance; (O) average of cystatin C‐ and proenkephalin‐A GFR versus CKD‐EPI formula‐based eGFR using plasma creatinine only. Graphs in red show pairs with poor correlation (*R* ^2^ ≤ 0.4); graphs in orange show pairs with moderate correlation (0.4 <*R* ^2^ ≤0.7) and graphs in green show pairs with good correlation (*R* ^2^ > 0.7). *p* < 0.05 for all the pairs.

**Figure S2.** Bland–Altman graphs assessing agreement between pairs of baseline unstimulated GFR measured by different methods. (A) CKD‐EPI formula‐based eGFR using both plasma creatinine and cystatin C versus proenkephalin‐A‐based eGFR by Beunders formula; (B) CKD‐EPI formula‐based eGFR using both plasma creatinine and cystatin C versus cystatin C‐based eGFR by Grubb formula; (C) CKD‐EPI formula‐based eGFR using both plasma creatinine and cystatin C versus creatinine clearance; (D) CKD‐EPI formula‐based eGFR using both plasma creatinine and cystatin C versus CKD‐EPI formula‐based eGFR using plasma creatinine only; (E) average of cystatin C‐ and proenkephalin‐A GFR versus CKD‐EPI formula‐based eGFR using both plasma creatinine and cystatin C; (F) average of cystatin C‐ and proenkephalin‐A GFR versus proenkephalin‐A‐based eGFR by Beunders formula; (G) average of cystatin C‐ and proenkephalin‐A GFR versus cystatin C‐based eGFR by Grubb formula; (H) average of cystatin C‐ and proenkephalin‐A GFR versus creatinine clearance; (I) average of cystatin C‐ and proenkephalin‐A GFR versus CKD‐EPI formula‐based eGFR using plasma creatinine only; (J) proenkephalin‐A‐based eGFR by Beunders formula versus CKD‐EPI formula‐based eGFR using plasma creatinine only; (K) cystatin C‐based eGFR by Grubb formula versus CKD‐EPI formula‐based eGFR using plasma creatinine only; (L) creatinine clearance versus CKD‐EPI formula‐based eGFR using plasma creatinine only; (M) proenkephalin‐A‐based eGFR by Beunders formula versus creatinine clearance; (N) cystatin C‐based eGFR by Grubb formula versus creatinine clearance; (O) cystatin C‐based eGFR by Grubb formula versus proenkephalin‐A‐based eGFR by Beunders formula. Limits of agreement are wider for those pairs including creatinine clearance.

**Figure S3.** Kidney functional reserve in healthy volunteers according to dietary protocol (*n* = 6 participants, *n* = 13 KFRs). (A) Plasma cystatin C, in mg/L; (B) Plasma PENK, in pmol/L; (C) Plasma creatinine, in μmol/L; (D) CysC‐eGFR, in mL/min/1.73 m^2^; (E) PENK‐eGFR, in mL/min/1.73 m^2^; (F) CrCl, in mL/min/1.73 m^2^. The *x*‐axis shows timepoints: baseline (before protein) and 1, 2, 3, 4 and 5 h after protein. Red: protocol 1 (low‐protein diet the day before + fasting overnight); green: protocol 2 (normal diet the day before + fasting overnight); and blue; protocol 3 (normal diet the day before + fasting for 2–4 h). Values depict standard least squares and confidence intervals. Mixed models were adjusted by timepoints, gender, age, baseline eGFR, BMI, KFR protocol and the interaction between KFR protocol and timepoints. Any preparatory diet and fasting protocols were similar for all of the markers.

**Figure S4.** Tubular Creatinine Secretion: (A) rate (mL/min); (B) mass (μmol/min), according to the dietary protocol (*n* = 6 participants, *n* = 13 KFRs). The rate of Cr secretion was calculated as CrCl minus eGFR[CysC /PENK] and the absolute (mass) Cr secretion from total Cr excretion (urine creatinine X urine volume) minus filtered creatinine {pCr concentration X eGFR[CysC /PENK]}. The *x*‐axis shows timepoints: baseline (before protein) and 1, 2, 3, 4, and 5 h after protein. Red: protocol 1 (low‐protein diet the day before + fasting overnight); green: protocol 2 (normal diet the day before + fasting overnight); and blue; protocol 3 (normal diet the day before + fasting for 2–4 h). Values depict standard least squares and confidence intervals. Mixed models were adjusted by timepoints, gender, age, baseline eGFR, BMI, KFR protocol and the interaction between KFR protocol and timepoints. Any preparatory diet and fasting protocols were similar for all of the markers.

**Figure S5.** Individual kidney functional reserve tests. (A–C) combined patients; (D–F) healthy participants; (G–I) cardiac surgery patients. The first column shows fractional CysC‐eGFR (normalized to baseline); the second fractional PENK‐eGFR (normalized to baseline), the third fractional creatinine clearance (normalized to baseline). The x‐axis shows time before (baseline) and 1, 2, 3, 4, and 5 h after protein.

**Figure S6.** Correlation between absolute kidney functional reserve measured by different methods. (A) absolute cystatin C‐based KFR versus absolute creatinine clearance‐based KFR; (B) absolute proenkephalin‐A‐based KFR versus absolute creatinine clearance‐based KFR; (C) absolute proenkephalin‐A‐based KFR versus absolute cystatin C‐based KFR. The graph in black shows a pair with no correlation (*p* > 0.05), the graph in red shows a pair with very poor correlation (*p* < 0.05) and the graph in orange shows a pair with better, but still poor, correlation (*p* < 0.05).

**Figure S7.** Correlation between fractional kidney functional reserve measured by different methods. (A) fractional cystatin C‐based KFR versus fractional creatinine clearance‐based KFR; (B) fractional proenkephalin‐A‐based KFR versus fractional creatinine clearance‐based KFR; (C) fractional proenkephalin‐A‐based KFR versus fractional cystatin C‐based KFR. Graphs in black show pairs with no correlation (*p* > 0.05) and the graph in red shows a pair with very poor correlation (*p* < 0.05).

**Figure S8.** Bland–Altman graphs assessing agreement between pairs of absolute and fractional kidney functional reserve measurements assessed by different methods. (A) absolute creatinine clearance‐based KFR versus absolute cystatin C‐based KFR; (B) absolute creatinine clearance‐based KFR versus absolute proenkephalin‐A‐based KFR; (C) absolute cystatin C‐based KFR versus absolute proenkephalin‐A‐based KFR; (D) fractional creatinine clearance‐based KFR versus fractional cystatin C‐based KFR; (E) fractional creatinine clearance‐based KFR versus fractional proenkephalin‐A‐based KFR; (F) fractional cystatin C‐based KFR versus fractional proenkephalin‐A‐based KFR. Limits of agreement are wider for those pairs including creatinine clearance‐based KFRs.

**Figure S9.** Correlation between KFR measured by different biomarkers and baseline kidney function by pCr‐eGFR (CKD‐EPI 2021). (A–C) Fractional KFR; (D–F) Absolute KFR. (A) CysC‐KFR (%) vs. baseline pCr‐eGFR (CKD‐EPI 2021); (B) PENK‐KFR (%) vs. baseline pCr‐eGFR (CKD‐EPI 2021); (C) CrCl‐KFR (%) vs. baseline pCr‐eGFR (CKD‐EPI 2021); (D) CysC‐KFR (in mL/min/1.73m^2^) vs. baseline pCr‐eGFR (CKD‐EPI 2021); (E) PENK‐KFR (in mL/min/1.73m^2^) vs. baseline pCr‐eGFR (CKD‐EPI 2021); (F) CrCl‐KFR (in mL/min/1.73m^2^) vs. baseline pCr‐eGFR (CKD‐EPI 2021).

**Figure S10.** Correlation between creatinine tubular secretion and baseline kidney function. (A) creatinine tubular secretion rate (mL/min) vs. baseline eGFR determined by eGFR [CysC/PENK]); (B.) creatinine tubular secretion mass (μmol/min) vs. baseline eGFR determined by eGFR [CysC/PENK]); (C) creatinine tubular secretion rate (mL/min) vs. baseline sCr‐eGFR (CKD‐EPI 2021); (D) creatinine tubular secretion mass (μmol/min) vs. baseline sCr‐eGFR (CKD‐EPI 2021). The rate of Cr secretion was calculated as CrCl minus eGFR[CysC /PENK] and absolute (mass) Cr secretion from total Cr excretion (urine creatinine × urine volume) minus filtered creatinine {pCr concentration × eGFR[CysC /PENK]}.

**Figure S11.** Kidney functional reserve, according to the use of tuna as Supplemental protein (*n* = 62). (A) Plasma cystatin C, in mg/L; (B) Plasma PENK, in pmol/L; (C) Plasma creatinine, in μmol/L; (D) UV Cr (Cr excreted in urine), in μmol/min; (E) CysC‐eGFR, in mL/min/1.73 m^2^; (F) PENK‐eGFR, in mL/min/1.73 m^2^; (G) CrCl, in mL/min/1.73 m^2^. Timepoints: baseline (before protein) and 1, 2, 3, 4, and 5 h after protein. Red: “no tuna”; Blue: 25 g tuna in addition to whey protein shake. Values represent standard least mean squares and confidence intervals. Mixed models were adjusted by timepoints, gender, age, baseline eGFR, BMI, KFR protocol, tuna as additional source of protein and the interaction between tuna protein and timepoints. Significance: *p < 0.05 vs. Baseline; #*p* < 0.05 for tuna as a fixed effect for the whole model; &*p* < 0.05 for interaction tuna × timepoints as a fixed effect for the whole model.

**Figure S12.** Creatinine tubular secretion: rate (A and C) and mass (B, D) (*n* = 62). (A and B) all individuals; (C and D) by the use of tuna as a Supplemental source of protein during KFR measurement. The x‐axis shows timepoints, represented by baseline (before protein) and 1, 2, 3, 4, and 5 h after protein. The rate of Cr secretion was calculated as CrCl minus eGFR[CysC /PENK] and absolute (mass) Cr secretion from total Cr excretion (urine creatinine × urine volume) minus filtered creatinine {pCr concentration × eGFR[CysC /PENK]}. Graphs depict standard least mean squares and confidence intervals. Red: “no tuna”; blue: 25 g tuna added to whey protein shake. Mixed models were adjusted by timepoints, gender, age, baseline eGFR, BMI, KFR protocol (C and D also were adjusted by tuna as additional source of protein and the interaction between tuna protein and timepoints). Significance: *p < 0.05 versus baseline; #*p* < 0.05 for tuna as a fixed effect for the whole model.

**Figure S13.** Fractional KFR and absolute KFR before and after kidney donation. (A) CysC‐KFR (%); (B) PENK‐KFR (%); (C) CrCl‐KFR (%); (D) CysC‐KFR (mL/min/1.73 m^2^); (E) PENK‐KFR (mL/min/1.73 m^2^); (F) CrCl‐KFR (mL/min/1.73 m^2^). Mixed models were adjusted by timepoints, gender, age, baseline eGFR, BMI, and KFR protocol. Significance: * *p* < 0.05 vs. Baseline; # *p* < 0.05 for time of the test as a fixed effect for the whole model.

**Table S1.** Baseline characteristics and adverse events according to preparatory diet and fasting time.
**Table S2.** KFR in healthy volunteers, according to preparatory diet and fasting time.

## Data Availability

The Excel data supporting the findings of this study are openly available in UNSWorks, the UNSW Open Access institutional repository at https://unsworks.unsw.edu.au/home (https://doi.org/10.26190/unsworks/31134).
