# Supplementary material for: Plasma proenkephalin‐a facilitates measurement of human kidney functional reserve: From healthy kidney donors to polymorbid patients
Source: Physiol Rep. 2026 Aug 26;14(16):e71083. doi: 10.14814/phy2.71083 (PMC13519117; doi:10.14814/phy2.71083)
Supplement: Supplementary file 1 [file PHY2-14-e71083-s008.docx]

**Supplemental information files**

**Supplemental Figures:**

Supplemental Figure 1: Correlation between baseline unstimulated glomerular filtration rate measured by different methods. A: Cystatin C-based eGFR by Grubb formula versus CKD-EPI formula-based eGFR using both plasma creatinine and cystatin C; B. Proenkephalin-A-based eGFR by Beunders formula versus CKD-EPI formula-based eGFR using both plasma creatinine and cystatin C; C. Proenkephalin-A-based eGFR by Beunders formula versus cystatin C-based eGFR by Grubb formula; D. Creatinine clearance versus CKD-EPI formula-based eGFR using both plasma creatinine and cystatin C; E. Creatinine clearance versus cystatin C-based eGFR by Grubb formula; F. Creatinine clearance versus proenkephalin-A-based eGFR by Beunders formula; G. CKD-EPI formula-based eGFR using plasma creatinine only versus CKD-EPI formula-based eGFR using both plasma creatinine and cystatin C; H. CKD-EPI formula-based eGFR using plasma creatinine only versus cystatin C-based eGFR by Grubb formula; I. CKD-EPI formula-based eGFR using plasma creatinine only versus proenkephalin-A-based eGFR by Beunders formula; J. CKD-EPI formula-based eGFR using plasma creatinine only versus creatinine clearance; K. average of cystatin C- and proenkephalin-A GFR versus CKD-EPI formula-based eGFR using both plasma creatinine and cystatin C; L. average of cystatin C- and proenkephalin-A GFR versus cystatin C-based eGFR by Grubb formula; M. average of cystatin C- and proenkephalin-A GFR versus proenkephalin-A-based eGFR by Beunders formula; N. average of cystatin C- and proenkephalin-A GFR versus creatinine clearance; O. average of cystatin C- and proenkephalin-A GFR versus CKD-EPI formula-based eGFR using plasma creatinine only. Graphs in red show pairs with poor correlation (R^2^ ≤ 0.4); graphs in orange show pairs with moderate correlation (0.4 < R^2^≤ 0.7) and graphs in green show pairs with good correlation (R^2^> 0.7). p< 0.05 for all the pairs.

Supplemental Figure 2: Bland-Altman graphs assessing agreement between pairs of baseline unstimulated GFR measured by different methods. A. CKD-EPI formula-based eGFR using both plasma creatinine and cystatin C versus proenkephalin-A-based eGFR by Beunders formula; B. CKD-EPI formula-based eGFR using both plasma creatinine and cystatin C versus cystatin C-based eGFR by Grubb formula; C. CKD-EPI formula-based eGFR using both plasma creatinine and cystatin C versus creatinine clearance; D. CKD-EPI formula-based eGFR using both plasma creatinine and cystatin C versus CKD-EPI formula-based eGFR using plasma creatinine only; E. average of cystatin C- and proenkephalin-A GFR versus CKD-EPI formula-based eGFR using both plasma creatinine and cystatin C; F. average of cystatin C- and proenkephalin-A GFR versus proenkephalin-A-based eGFR by Beunders formula; G. average of cystatin C- and proenkephalin-A GFR versus cystatin C-based eGFR by Grubb formula; H. average of cystatin C- and proenkephalin-A GFR versus creatinine clearance; I. average of cystatin C- and proenkephalin-A GFR versus CKD-EPI formula-based eGFR using plasma creatinine only; J. proenkephalin-A-based eGFR by Beunders formula versus CKD-EPI formula-based eGFR using plasma creatinine only; K. cystatin C-based eGFR by Grubb formula versus CKD-EPI formula-based eGFR using plasma creatinine only; L. creatinine clearance versus CKD-EPI formula-based eGFR using plasma creatinine only; M. proenkephalin-A-based eGFR by Beunders formula versus creatinine clearance; N. cystatin C-based eGFR by Grubb formula versus creatinine clearance; O. cystatin C-based eGFR by Grubb formula versus proenkephalin-A-based eGFR by Beunders formula. Limits of agreement are wider for those pairs including creatinine clearance.

Supplemental Figure 3: Kidney functional reserve in healthy volunteers according to dietary protocol (n=6 participants, n=13 KFRs). A. Plasma cystatin C, in mg/L; B. Plasma PENK, in pmol/L; C. Plasma creatinine, in umol/L; D. CysC-eGFR, in ml/min/1.73m2; E. PENK-eGFR, in ml/min/1.73m2; F. CrCl, in ml/min/1.73m2. The x-axis shows timepoints: baseline (before protein) and 1, 2, 3, 4 and 5 hours after protein. Red: protocol 1 (low-protein diet the day before + fasting overnight); green: protocol 2 (normal diet the day before + fasting overnight); and blue; protocol 3 (normal diet the day before + fasting for 2-4 hours). Values depict standard least squares and confidence intervals. Mixed models were adjusted by timepoints, gender, age, baseline eGFR, BMI, KFR protocol and the interaction between KFR protocol and timepoints. Any preparatory diet and fasting protocols were similar for all of the markers.

Exact p-values versus Baseline:

A. CysC (mg/L), by KFR protocol: 1. Low-protein diet + fasting for 8-12 hours: T1: p=0.9921; T2: p=0.9989; T3: p=0.8448; T4: p=0.8728; T5: p=0.9258 / 2. Normal diet + fasting for 8-12 hours: T1: p=1.0000; T2: p=0.9291; T3: p=0.8970; T4: p=0.9528; T5: p=0.9999 / 3. Normal diet + fasting for 2-4 hours: T1: p=0.9779; T2: p=0.9536; T3: p=0.5008; T4: p=0.6454; T5: p=0.8963. KFR protocol as a fixed effect for the whole model: p=0.0476; interaction KFR protocol x timepoints as a fixed effect for the whole model: p=0.9968.
B. PENK (pmol/L), by KFR protocol: 1. Low-protein diet + fasting for 8-12 hours: T1: p=0.1303; T2: p=0.1275; T3: p=0.2824; T4: p=0.7449; T5: p=0.9714 / 2. Normal diet + fasting for 8-12 hours: T1: p=0.1271; T2: p=0.0586; T3: p=0.2164; T4: p=0.9039; T5: p=0.9562 / 3. Normal diet + fasting for 2-4 hours: T1: p=0.4750; T2: p=0.3371; T3: p=0.4113; T4: p=0.7886; T5: p=0.9990. KFR protocol as a fixed effect for the whole model: p=0.7274; interaction KFR protocol x timepoints as a fixed effect for the whole model: p=0.9999.
C. pCr (umol/L), by KFR protocol: 1. Low-protein diet + fasting for 8-12 hours: T1: p=0.2422; T2: p=0.1596; T3: p=0.2205; T4: p=0.0035; T5: p=0.0289 / 2. Normal diet + fasting for 8-12 hours: T1: p=1.0000; T2: p=1.0000; T3: p=0.3776; T4: p=0.0374; T5: p=0.0374 / 3. Normal diet + fasting for 2-4 hours: T1: p=1.0000; T2: p=0.7543; T3: p=0.3298; T4: p=0.1113; T5: p=0.0921. KFR protocol as a fixed effect for the whole model: p=0.2612; interaction KFR protocol x timepoints as a fixed effect for the whole model: p=0.8888.
D. CysC-eGFR (ml/min/1.73m2), by KFR protocol: 1. Low-protein diet + fasting for 8-12 hours: T1: p=0.9837; T2: p=0.9990; T3: p=0.8845; T4: p=0.9115; T5: p=0.9518 / 2. Normal diet + fasting for 8-12 hours: T1: p=1.0000; T2: p=0.9929; T3: p=0.9539; T4: p=0.9951; T5: p=1.0000 / 3. Normal diet + fasting for 2-4 hours: T1: p=0.9916; T2: p=0.9940; T3: p=0.6479; T4: p=0.7927; T5: p=0.9625. KFR protocol as a fixed effect for the whole model: p=0.0402; interaction KFR protocol x timepoints as a fixed effect for the whole model: p=0.9979.
E. PENK-eGFR (ml/min/1.73m2), by KFR protocol: 1. Low-protein diet + fasting for 8-12 hours: T1: p=0.2854; T2: p=0.2308; T3: p=0.5797; T4: p=0.9626; T5: p=0.9994 / 2. Normal diet + fasting for 8-12 hours: T1: p=0.2174; T2: p=0.0822; T3: p=0.3473; T4: p=0.9795; T5: p=0.9997 / 3. Normal diet + fasting for 2-4 hours: T1: p=0.8198; T2: p=0.6865; T3: p=0.7984; T4: p=0.9636; T5: p=1.0000. KFR protocol as a fixed effect for the whole model: p=0.4650; interaction KFR protocol x timepoints as a fixed effect for the whole model: p=1.0000.
F. CrCl (ml/min/1.73m2), by KFR protocol: 1. Low-protein diet + fasting for 8-12 hours: T1: p=0.9999; T2: p=1.0000; T3: p=1.0000; T4: p=1.0000; T5: p=0.9999 / 2. Normal diet + fasting for 8-12 hours: T1: p=1.0000; T2: p=0.9032; T3: p=0.9998; T4: p=0.9285; T5: p=1.0000 / 3. Normal diet + fasting for 2-4 hours: T1: p=1.0000; T2: p=1.0000; T3: p=0.9999; T4: p=1.0000; T5: p=0.9977. KFR protocol as a fixed effect for the whole model: p=0.8125; interaction KFR protocol x timepoints as a fixed effect for the whole model: p=0.9685.

Supplemental Figure 4: Tubular Creatinine Secretion: A. rate (ml/min); B. mass (umol/min), according to the dietary protocol (n=6 participants, n=13 KFRs). The rate of Cr secretion was calculated as CrCl minus eGFR[CysC /PENK] and the absolute (mass) Cr secretion from total Cr excretion (urine creatinine X urine volume) minus filtered creatinine {pCr concentration X eGFR[CysC /PENK]}. The x-axis shows timepoints: baseline (before protein) and 1, 2, 3, 4 and 5 hours after protein. Red: protocol 1 (low-protein diet the day before + fasting overnight); green: protocol 2 (normal diet the day before + fasting overnight); and blue; protocol 3 (normal diet the day before + fasting for 2-4 hours). Values depict standard least squares and confidence intervals. Mixed models were adjusted by timepoints, gender, age, baseline eGFR, BMI, KFR protocol and the interaction between KFR protocol and timepoints. Any preparatory diet and fasting protocols were similar for all of the markers.

Exact p-values versus Baseline:

A. Cr tubular secretion rate: CrCl minus eGFR [CysC/PENK] (ml/min), by KFR protocol: 1. Low-protein diet + fasting for 8-12 hours: T1: p=0.2157; T2: p=0.8582; T3: p=0.4551; T4: p=0.8731; T5: p=1.0000 / 2. Normal diet + fasting for 8-12 hours: T1: p=0.8203; T2: p=0.9802; T3: p=0.9934; T4: p=1.0000; T5: p=1.0000 / 3. Normal diet + fasting for 2-4 hours: T1: p=0.8653; T2: p=0.9943; T3: p=0.3355; T4: p=0.8140; T5: p=0.8186. KFR protocol as a fixed effect for the whole model: p=0.9326; interaction KFR protocol x timepoints as a fixed effect for the whole model: p=0.7519.
B. Absolute Cr tubular secretion: UV Cr minus filtered Cr (umol/min), by KFR protocol: 1. Low-protein diet + fasting for 8-12 hours: T1: p=0.1637; T2: p=0.9057; T3: p=0.6857; T4: p=0.8233; T5: p=1.0000 / 2. Normal diet + fasting for 8-12 hours: T1: p=0.6357; T2: p=0.9580; T3: p=0.9292; T4: p=1.0000; T5: p=0.9990 / 3. Normal diet + fasting for 2-4 hours: T1: p=0.8961; T2: p=0.9977; T3: p=0.4373; T4: p=0.7546; T5: p=0.8565. KFR protocol as a fixed effect for the whole model: p=0.8219; interaction KFR protocol x timepoints as a fixed effect for the whole model: p=0.8015.

Supplemental Figure 5: Individual kidney functional reserve tests. A-C: combined patients; D-F: healthy participants; G-I: cardiac surgery patients. The first column shows fractional CysC-eGFR (normalised to baseline); the second fractional PENK-eGFR (normalised to baseline), the third fractional creatinine clearance (normalised to baseline). The x-axis shows time before (baseline) and 1, 2, 3, 4 and 5 hours after protein.

Supplemental Figure 6: Correlation between absolute kidney functional reserve measured by different methods. A: absolute cystatin C-based KFR versus absolute creatinine clearance-based KFR; B. absolute proenkephalin-A-based KFR versus absolute creatinine clearance-based KFR; C. absolute proenkephalin-A-based KFR versus absolute cystatin C-based KFR. The graph in black shows a pair with no correlation (p>0.05), the graph in red shows a pair with very poor correlation (p< 0.05) and the graph in orange shows a pair with better, but still poor, correlation (p< 0.05).

Supplemental Figure 7: Correlation between fractional kidney functional reserve measured by different methods. A: fractional cystatin C-based KFR versus fractional creatinine clearance-based KFR; B. fractional proenkephalin-A-based KFR versus fractional creatinine clearance-based KFR; C. fractional proenkephalin-A-based KFR versus fractional cystatin C-based KFR. Graphs in black show pairs with no correlation (p>0.05) and the graph in red shows a pair with very poor correlation (p< 0.05).

Supplemental Figure 8: Bland-Altman graphs assessing agreement between pairs of absolute and fractional kidney functional reserve measurements assessed by different methods. A. absolute creatinine clearance-based KFR versus absolute cystatin C-based KFR; B. absolute creatinine clearance-based KFR versus absolute proenkephalin-A-based KFR; C. absolute cystatin C-based KFR versus absolute proenkephalin-A-based KFR; D. fractional creatinine clearance-based KFR versus fractional cystatin C-based KFR; E. fractional creatinine clearance-based KFR versus fractional proenkephalin-A-based KFR; F. fractional cystatin C-based KFR versus fractional proenkephalin-A-based KFR. Limits of agreement are wider for those pairs including creatinine clearance-based KFRs.

Supplemental Figure 9: Correlation between KFR measured by different biomarkers and baseline kidney function by pCr-eGFR (CKD-EPI 2021). A-C: Fractional KFR; D-F: Absolute KFR. A. CysC-KFR (%) vs baseline pCr-eGFR (CKD-EPI 2021); B. PENK-KFR (%) vs baseline pCr-eGFR (CKD-EPI 2021); C. CrCl-KFR (%) vs baseline pCr-eGFR (CKD-EPI 2021); D. CysC-KFR (in ml/min/1.73m^2^) vs baseline pCr-eGFR (CKD-EPI 2021); E. PENK-KFR (in ml/min/1.73m^2^) vs baseline pCr-eGFR (CKD-EPI 2021); F. CrCl-KFR (in ml/min/1.73m^2^) vs baseline pCr-eGFR (CKD-EPI 2021).

Supplemental Figure 10: Correlation between creatinine tubular secretion and baseline kidney function. A. creatinine tubular secretion rate (ml/min) vs baseline eGFR determined by eGFR [CysC/PENK]); B. creatinine tubular secretion mass (umol/min) vs baseline eGFR determined by eGFR [CysC/PENK]); C. creatinine tubular secretion rate (ml/min) vs baseline sCr-eGFR (CKD-EPI 2021); D. creatinine tubular secretion mass (umol/min) vs baseline sCr-eGFR (CKD-EPI 2021). The rate of Cr secretion was calculated as CrCl minus eGFR[CysC /PENK] and absolute (mass) Cr secretion from total Cr excretion (urine creatinine X urine volume) minus filtered creatinine {pCr concentration X eGFR[CysC /PENK]}.

Supplemental Figure 11: Kidney functional reserve, according to the use of tuna as Supplemental protein (n=62). A. Plasma cystatin C, in mg/L; B. Plasma PENK, in pmol/L; C. Plasma creatinine, in umol/L; D. UV Cr (Cr excreted in urine), in umol/min; E. CysC-eGFR, in ml/min/1.73m2; F. PENK-eGFR, in ml/min/1.73m2; G. CrCl, in ml/min/1.73m2. Timepoints: baseline (before protein) and 1, 2, 3, 4 and 5 hours after protein. Red: ‘no tuna’; Blue: 25g tuna in addition to whey protein shake. Values represent standard least mean squares and confidence intervals. Mixed models were adjusted by timepoints, gender, age, baseline eGFR, BMI, KFR protocol, tuna as additional source of protein and the interaction between tuna protein and timepoints. Significance: * p < 0.05 vs Baseline; # p<0.05 for tuna as a fixed effect for the whole model; & p<0.05 for interaction tuna x timepoints as a fixed effect for the whole model.

Exact p-values versus Baseline:

A. CysC (mg/L), by tuna protein: No tuna: T1: p=0.2335; T2: p=0.0485; T3: p=0.0075; T4: p=0.0208; T5: p=0.0213 / Tuna: T1: p=0.9935; T2: p=0.9887; T3: p=0.9691; T4: p=0.7965; T5: p=0.7723. Tuna protein as a fixed effect for the whole model: p=0.5448; interaction tuna protein x timepoints as a fixed effect for the whole model: p=0.5159.
B. PENK (pmol/L), by tuna protein: No tuna: T1: p<0.0001; T2: p<0.0001; T3: p<0.0001; T4: p<0.0001; T5: p<0.0001 / Tuna: T1: p=0.4816; T2: p=0.2668; T3: p=0.0924; T4: p=0.9355; T5: p=0.9959. Tuna protein as a fixed effect for the whole model: p=0.1740; interaction tuna protein x timepoints as a fixed effect for the whole model: p=0.4562.
C. pCr (umol/L), by tuna protein: No tuna: T1: p=0.9924; T2: p=0.9997; T3: p=0.3259; T4: p=0.0338; T5: p=0.0039 / Tuna: T1: p=0.1802; T2: p=0.0546; T3: p=0.0199; T4: p=0.0083; T5: p=0.0611. Tuna protein as a fixed effect for the whole model: p=0.2417; interaction tuna protein x timepoints as a fixed effect for the whole model: p=0.0053.
D. UV Cr (umolmin), by tuna protein: No tuna: T1: p=0.7720; T2: p=0.9721; T3: p=0.9974; T4: p=1.0000; T5: p=1.0000 / Tuna: T1: p=0.7494; T2: p=0.1291; T3: p=1.0000; T4: p=0.0064; T5: p=0.5296. Tuna protein as a fixed effect for the whole model: p=0.0480; interaction tuna protein x timepoints as a fixed effect for the whole model: p=0.0157.
E. CysC-eGFR (ml/min/1.73m2), by tuna protein: No tuna: T1: p=0.0306; T2: p=0.0015; T3: p<0.0005; T4: p=0.0015; T5: p=0.0055 / Tuna: T1: p=0.9020; T2: p=0.9995; T3: p=1.0000; T4: p=1.0000; T5: p=1.0000. Tuna protein as a fixed effect for the whole model: p=0.6029; interaction tuna protein x timepoints as a fixed effect for the whole model: p=0.5968.
F. PENK-eGFR (ml/min/1.73m2), by tuna protein: No tuna: T1: p<0.0005; T2: p<0.0005; T3: p<0.0005; T4: p<0.0005; T5: p<0.0005 / Tuna: T1: p=0.2684; T2: p=0.2392; T3: p=0.2854; T4: p=0.5798; T5: p=0.8649. Tuna protein as a fixed effect for the whole model: p=0.2686; interaction tuna protein x timepoints as a fixed effect for the whole model: p=0.7667.
G. CrCl (ml/min/1.73m2), by tuna protein: No tuna: T1: p=0.8575; T2: p=0.7845; T3: p=0.7170; T4: p=0.9513; T5: p=0.8205 / Tuna: T1: p=0.5971; T2: p=0.3942; T3: p=1.000; T4: p=0.3605; T5: p=0.8620. Tuna protein as a fixed effect for the whole model: p=0.1611; interaction tuna protein x timepoints as a fixed effect for the whole model: p=0.1799.

Supplemental Figure 12: Creatinine tubular secretion: rate (A and C) and mass (B, D) (n=62). A and B: all individuals; C and D: by the use of tuna as a Supplemental source of protein during KFR measurement. The x-axis shows timepoints, represented by baseline (before protein) and 1, 2, 3, 4 and 5 hours after protein. The rate of Cr secretion was calculated as CrCl minus eGFR[CysC /PENK] and absolute (mass) Cr secretion from total Cr excretion (urine creatinine X urine volume) minus filtered creatinine {pCr concentration X eGFR[CysC /PENK]}. Graphs depict standard least mean squares and confidence intervals. Red: ‘no tuna’; blue: 25g tuna added to whey protein shake. Mixed models were adjusted by timepoints, gender, age, baseline eGFR, BMI, KFR protocol (C and D also were adjusted by tuna as additional source of protein and the interaction between tuna protein and timepoints). Significance: * p<0.05 versus baseline; # p<0.05 for tuna as a fixed effect for the whole model.

Exact p-values versus Baseline:

A. Cr tubular secretion rate: CrCl minus eGFR [CysC/PENK] (ml/min): T1: p=0.0014; T2: p=0.0752; T3: p=0.0130; T4: p=0.6867; T5: p=0.9911.
B. Absolute Cr tubular secretion: UV Cr minus filtered Cr (umol/min): T1: p=0.0024; T2: p=0.1144; T3: p=0.0205; T4: p=0.7895; T5: p=0.9184.
C. Cr tubular secretion rate: CrCl minus eGFR [CysC/PENK] (ml/min), by tuna protein: No tuna: T1: p=0.0005; T2: p=0.0532; T3: p=0.0299; T4: p=0.5946; T5: p=0.9961 / Tuna: T1: p=0.9521; T2: p=0.8280; T3: p=0.9982; T4: p=0.5253; T5: p=0.9509. Tuna protein as a fixed effect for the whole model: p=0.0841; interaction tuna protein x timepoints as a fixed effect for the whole model: p=0.1643.
D. Absolute Cr tubular secretion: UV Cr minus filtered Cr (umol/min), by tuna protein: No tuna: T1: p=0.0012; T2: p=0.0784; T3: p=0.0567; T4: p=0.5110; T5: p=0.9422 / Tuna: T1: p=0.9973; T2: p=0.7067; T3: p=0.9828; T4: p=0.0660; T5: p=0.9122. Tuna protein as a fixed effect for the whole model: p=0.0363; interaction tuna protein x timepoints as a fixed effect for the whole model: p=0.0299.

Supplemental Figure 13: Fractional KFR and absolute KFR before and after kidney donation. A. CysC-KFR (%); B. PENK-KFR (%); C. CrCl-KFR (%); D. CysC-KFR (ml/min/1.73m2); E. PENK-KFR (ml/min/1.73m2); F. CrCl-KFR (ml/min/1.73m2). Mixed models were adjusted by timepoints, gender, age, baseline eGFR, BMI and KFR protocol. Significance: * p < 0.05 vs Baseline; # p <0.05 for time of the test as a fixed effect for the whole model.

Exact p-values versus Baseline:

A. CysC-KFR (%): 1 m-postoperative: p=0.1394; 3 m-postoperative: p=0.9343. Time of the test as a fixed effect for the whole model: p=0.1915
B. PENK-KFR (%): 1 m-postoperative: p=0.9867; 3 m-postoperative: p=0.8273. Time of the test as a fixed effect for the whole model: p=0.8734
C. CrCl-KFR (%): 1 m-postoperative: p=0.3908; 3 m-postoperative: p=0.9648. Time of the test as a fixed effect for the whole model: p=0.4890
D. CysC-KFR (ml/min/1.73m2): 1 m-postoperative: p=0.0019; 3 m-postoperative: p=0.2157. Time of the test as a fixed effect for the whole model: p=0.0043
E. PENK-KFR (ml/min/1.73m2): 1 m-postoperative: p=0.0240; 3 m-postoperative: p=0.0073. Time of the test as a fixed effect for the whole model: p=0.0148
F. CrCl-KFR (ml/min/1.73m2): 1 m-postoperative: p= 0.0148; 3 m-postoperative: p=0.1646. Time of the test as a fixed effect for the whole model: p=0.0240

**Supplemental** **Tables:**

**Supplemental Table 1: Baseline Characteristics and Adverse Events according to preparatory diet and fasting time.**

|  | **All** | **Healthy participants** | **Cardiac surgery** | **p-value** | **n** |
| --- | --- | --- | --- | --- | --- |
| **Preoperative KFR tests** | **n=62** | **n=22** | **n=40** |  |  |
| *Participants:* |  |  |  | <0.001 | 62 |
| Healthy volunteers, n (%) | 6 (9.68) | 6 (27.3) | 0 |  |  |
| Kidney donors, n (%) | 14 (22.6) | 14 (63.6) | 0 |  |  |
| Autotransplantantion, n(%) | 2 (3.23) | 2 (9.1) | 0 |  |  |
| Cardiac surgery, n (%) | 40 (64.5) | 0 | 40 (100) |  |  |
| **KFR protocol** |  |  |  |  |  |
| *Fasting and diet:* |  |  |  | 0.027 | 62 |
| low-protein diet + fasting overnight, n (%) | 10 (16.1) | 5 (22.7) | 5 (12.5) |  |  |
| normal diet + fasting overnight, n (%) | 30 (48.4) | 14 (63.6) | 16 (40) |  |  |
| normal diet + fasting 2-4h  n (%) | 22 (35.5) | 3 (13.6) | 19 (47.5) |  |  |
| **Supplemental Protein** |  |  |  |  |  |
| Tuna,  n (%) | 7 (11.3) | 1 (4.5) | 6 (15) | 0.405 | 62 |
| Nuts,  n (%) | 7 (11.3) | 1 (4.5) | 6 (15) | 0.405 | 62 |
| **Adverse Events** |  |  |  |  |  |
| All protein tolerated, n (%) | 61 (98.4) | 22 (100) | 39 (97.5) | 1 | 62 |
| Nausea, n (%) | 12 (19.4) | 4 (18.2) | 8 (20) | 1 | 62 |
| Diarrhoea, n (%) | 4 (6.45) | 0 (0) | 4 (10) | 0.287 | 62 |

**Supplemental Table 2: KFR in healthy volunteers, according to preparatory diet and fasting time.**

| **KFR measurements** | **All** | **Protocol 1** | **Protocol 2** | **Protocol 3** | **p-value** | **n** |
| --- | --- | --- | --- | --- | --- | --- |
|  | **n=13** | **n=4** | **n=6** | **n=3** |  |  |
| Fractional CysC-KFR (%),  median [IQR] | 8.52 ± 5.81 | 8.03 ± 5.43 | 7.03 ± 6.18 | 12.2 ± 5.99 | 0.489 | 13 |
| Absolute CysC-KFR (ml/min/1.73m^2^), median [IQR] | 8.78 ± 6.02 | 8.71 ± 6.34 | 7.08 ± 5.59 | 12.2 ± 7.23 | 0.52 | 13 |
| Fractional PENK-based KFR (%), median [IQR] | 48.6 ± 24.4 | 57.8 ± 30.9 | 47.6 ± 20.2 | 38.0 ± 28.0 | 0.607 | 13 |
| Absolute PENK-based KFR (ml/min/1.73m^2^), median [IQR] | 36.3 ± 17.5 | 42.3 ± 19.3 | 37.0 ± 17.8 | 26.8 ± 16.7 | 0.546 | 13 |
| Fractional CrCl-based KFR (%), median [IQR] | 14.0 [0.30;18.3] | 9.00  [-0.52;22.8] | 13.6 [3.47;24.5] | 14.0 [3.55;16.1] | 0.83 | 13 |
| Absolute CrCl-based KFR (ml/min/1.73m^2^), median [IQR] | 15.8 ± 20.0 | 16.7 ± 22.3 | 18.6 ± 22.7 | 9.07 ± 16.7 | 0.822 | 13 |
